# Verrucomicrobial methanotrophs grow on diverse C3 compounds and use a homolog of particulate methane monooxygenase to oxidize acetone

**DOI:** 10.1038/s41396-021-01037-2

**Published:** 2021-06-22

**Authors:** Samuel Imisi Awala, Joo-Han Gwak, Yong-Man Kim, So-Jeong Kim, Andrea Strazzulli, Peter F. Dunfield, Hyeokjun Yoon, Geun-Joong Kim, Sung-Keun Rhee

**Affiliations:** 1 Department of Biological Sciences and Biotechnology, Chungbuk National University, 1 Chungdae-ro, Seowon-Gu, Cheongju, 28644 Republic of Korea; 2 Geologic Environment Research Division, Korea Institute of Geoscience and Mineral Resources, Daejeon, 34132 Republic of Korea; 3 Department of Biology, University of Naples “Federico II”, Complesso Universitario Di Monte S. Angelo, Via Cupa Nuova Cinthia 21, 80126 Naples, Italy; 4 Department of Biological Sciences, University of Calgary, 2500 University Dr. NW, Calgary, AB T2N 1N4 Canada; 5 Biological and Genetic Resources Assessment Division, National Institute of Biological Resources, 42 Hwangyeong-ro, Seo-gu, Incheon, 22689 Republic of Korea; 6 Department of Biological Sciences and Research Center of Ecomimetics, College of Natural Sciences, Chonnam National University, Yongbong-ro, Buk-gu, Gwangju, 61186 Republic of Korea

**Keywords:** Environmental microbiology, Biogeochemistry, Microbial ecology

## Abstract

Short-chain alkanes (SCA; C2-C4) emitted from geological sources contribute to photochemical pollution and ozone production in the atmosphere. Microorganisms that oxidize SCA and thereby mitigate their release from geothermal environments have rarely been studied. In this study, propane-oxidizing cultures could not be grown from acidic geothermal samples by enrichment on propane alone, but instead required methane addition, indicating that propane was co-oxidized by methanotrophs. “Methylacidiphilum” isolates from these enrichments did not grow on propane as a sole energy source but unexpectedly did grow on C3 compounds such as 2-propanol, acetone, and acetol. A gene cluster encoding the pathway of 2-propanol oxidation to pyruvate via acetol was upregulated during growth on 2-propanol. Surprisingly, this cluster included one of three genomic operons (*pmoCAB3*) encoding particulate methane monooxygenase (PMO), and several physiological tests indicated that the encoded PMO3 enzyme mediates the oxidation of acetone to acetol. Acetone-grown resting cells oxidized acetone and butanone but not methane or propane, implicating a strict substrate specificity of PMO3 to ketones instead of alkanes. Another PMO-encoding operon, *pmoCAB2*, was induced only in methane-grown cells, and the encoded PMO2 could be responsible for co-metabolic oxidation of propane to 2-propanol. In nature, propane probably serves primarily as a supplemental growth substrate for these bacteria when growing on methane.

## Introduction

Short-chain alkanes (SCA) such as ethane and propane released to the atmosphere play a significant role in ozone formation and tropospheric photochemical pollution. Geological emissions of propane account for at least 10% of total global emissions, a comparable amount to other natural sources [1, 2]. Geological sources of propane include mud volcanoes, seeps, and geothermal areas, although microbial hydrocarbon oxidizers in these areas act as biofilters to mitigate total emissions [1–3]. Aerobic propane-degrading bacteria belonging mostly to the phylum Actinobacteria have been described from various environments [4, 5]. Oxidation of gaseous hydrocarbons, including propane, is also performed by strictly anaerobic sulfate-reducing bacteria in marine hydrocarbon seep environments [6, 7]. However, studies on aerobic microbial propane oxidation in geothermal environments are rare [8]. In contrast, aerobic methane oxidation mediated by verrucomicrobial methanotrophs has been widely investigated in acidic geothermal environments [9–13].

Copper membrane monooxygenase (CuMMO) is a key enzyme involved in the biogeochemical cycles of carbon and nitrogen [14–19]. There are three different primary substrates of CuMMO known so far: (1) methane for particulate methane monooxygenase (PMO), (2) short-chain alkanes including propane for hydrocarbon monooxygenase (HMO), and (3) ammonia for ammonia monooxygenase (AMO). Microorganisms employing CuMMO can be important aerobic oxidizers of gaseous hydrocarbons in various environments [14, 20]. Although CuMMOs are involved in the initial oxidation step of these substrates, they are promiscuous in their catalytic substrates [21–24]. For example, PMO-containing methanotrophs are known to co-metabolically oxidize a wide range of alkanes, halogenated alkanes, and alkenes; although they produce metabolic energy only via methane oxidation [8, 25]. In addition, some facultative methylotrophs such as *Methylocella* spp., which possess promiscuous diiron monooxygenases instead of PMO, utilize propane as well as methane since they have pathways for utilization of the propanol and methanol [3, 26, 27]. Thus, the presence or absence of metabolic pathways further degrading oxygenated substrates determines the substrate utilization capability of CuMMO-encoding bacteria.

Within the last two decades, highly divergent copies of the CuMMO-encoding operon have been discovered in the proteobacterial [16, 28, 29] and verrucomicrobial methanotrophs [9, 13, 30]. These divergent CuMMO-encoding operons are often hypothesized to have distinct physiological roles. The two *pmo* operons in *Methylocystis* sp. strain SC2 were demonstrated to possess varying affinities for methane. In this strain, the *pmoCAB1* was found to function only at high methane mixing ratios (above 600 ppmv), whereas the *pmoCAB*2 was involved in methane oxidation at the much lower atmospheric level (1.75 ppmv) [31]. The function and substrate specificity of other forms of the CuMMO, such as those encoded by the *pxmABC* found in some proteobacterial methanotrophs, remain unelucidated, although increased transcription of the *pxmABC* operon under hypoxia has been reported [29, 32]. The genomes of most verrucomicrobial methanotrophs in the proposed genus “Methylacidiphilum” possess three divergent operons encoding CuMMO [33–35], although there are exceptions. For example, “Methylacidiphilum sp.” strain Yel was reported to possess only a single complete *pmoCAB*3 operon based on analysis of its draft genome [36, 37]. RNA-sequencing results revealed that the expression levels of the *pmoCAB1-2* operons in various “Methylacidiphilum” strains were tightly regulated in response to oxygen concentration, whereas the expression level of the most divergent paralog, *pmoCAB3*, was very low or undetectable under methanotrophic growth conditions [38–40]. Recently, upregulation of *pmoCAB3* in “Methylacidiphilum fumariolicum” SolV and “Methylacidiphilum sp.” RTK17.1 was observed in chemostat cultures grown on a mixture of methanol and propane [8] or on formic acid in the absence of methane [41], respectively. These results suggest that PMO3 plays some role in alkane degradation, although the precise nature of this role is not clear. Other CuMMO-encoding genes with unknown functions are widespread in various microbial clades: e.g., *Smaragdicoccus niigatensis* DSM 44881, *Polaromonas* spp., *Rhodoferax* spp., *Solimonas aquatica* DSM 25927, and *Rhodomicrobium* spp. [15, 42–44]. Since CuMMOs are highly promiscuous, there are possibilities for extending the known natural substrates of CuMMOs.

This study investigated SCA oxidation in acidic geothermal samples obtained from the Pisciarelli hot spring, Italy. Natural gas released from this site is usually a mixture of methane, ethane, propane, and butane [45]. Hence, we aimed at determining the microorganisms involved in mitigating SCA release from this environment. From our results, propane was co-metabolically consumed only in the presence of methane in samples highly enriched with “Methylacidiphilum” cells. “Methylacidiphilum” isolates from these enrichments did not grow solely on propane but surprisingly did utilize C3 compounds produced as intermediates of propane oxidation (e.g., 2-propanol, acetone, acetol) via a complete oxidation pathway encoded by the isolates. A novel function of the CuMMO encoded by *pmoCAB3* as an acetone monooxygenase (AcMO) was revealed.

## Materials and methods

### Environmental sampling

During a field campaign in September 2018, samples of mud/water mixtures were taken from two different ponds at the Pisciarelli hot spring (40°49′45.1′′N 14°08′49.7′′E) in Pozzuoli, Italy. The Pisciarelli fumarolic field is located outside of the Solfatara crater, and the fumarolic gases are composed mainly of water, followed by carbon dioxide, hydrogen sulfide, dinitrogen, dihydrogen, methane, helium, argon, carbon monoxide, and SCA [45–48]. In addition to methane (55–115 ppmv), a mixture of SCAs including ethane (820–1847 ppbv), propane (70–168 ppbv), and butane (5–38 ppbv) are released from this site [45]. Temperatures can reach as hot as 100–110 °C. The detailed geochemical characteristics of this field have been described previously [45–48]. Surface water and mud samples (at a depth of 10 cm) were taken 50–100 cm from the shores of the fumarole ponds. Temperature and pH were measured in situ with an HI-93510 thermometer (HANNA instruments, Padova, Italy) equipped with a Pt100 probe and a pH meter for field use (sensION+PH1 equipped with 5051T electrode (HACH)). The selected sites for sampling had temperatures between 30 and 95 °C and pH values between 1.5 and 7.0. The samples were transferred into sterile 50-ml plastic tubes and stored at 4 °C before use.

### Enrichment and isolation

Enrichment was performed using a low-salt mineral (LSM) medium that contained 0.4 mM MgSO_4_·7H_2_O, 0.2 mM K_2_SO_4_, and 0.1 mM CaCl_2_·2H_2_O. Upon sterilization of the basal salt solution, 1 mM (NH_4_)_2_SO_4_, 0.1 mM KH_2_PO_4_, 1 µM CeCl_3_, 1 µM LaCl_3_, 1 ml vitamin solution, 1 ml trace elements solution 1 (TES1), and 1 ml trace elements solution 2 (TES2) (see the detailed composition of vitamin and trace elements solutions in Table S1) were added as 0.1 µm-filter-sterilized solutions unless otherwise stated. A series of the LSM media with varying pH values (pH 2.0, 4.5, and 6.5) were made with 2 mM sulfuric acid (pH 2) and 5 mM 4-morpholineethanesulfonic acid (pH 4.5 and 6.5). The enrichment was started by adding 2 ml of sample slurry to 20 ml of LSM medium in sterile 160-ml serum vials capped with blue butyl rubber stoppers. The headspaces of the sealed bottles contained CO_2_ (5% v/v) as a carbon source and a series of gas substrates as energy sources. The following combination of gas substrates (v/v) were used; (i) 5% methane (ii) 5% propane (iii) 5% each of methane and propane. These bottles were incubated at 42, 55, and 65 °C with shaking (150 rpm) for 4–8 weeks. To determine substrate consumption in these incubations, 100 µl of headspace gas was sampled from each bottle using a syringe and analyzed on a GC-2010 Plus gas chromatograph (see below for details). Methane and propane oxidation were observed in some of the setups, and 2 ml amounts of these cultures were transferred to fresh media supplied with the same gas compositions.

The microorganisms involved in methane and propane oxidation in the enriched cultures at pH 4.5 at 55 °C were isolated via repeated dilution to extinction. Following several dilutions of the cultures into fresh liquid media, cultures were filtered through 0.2-µm polycarbonate filters (Whatman, UK) [49] and also spread on LSM media plates solidified with phytagel (Sigma-Aldrich, Korea) at pH 4.5. The filters were placed on the liquid LSM medium (pH 4.5) in Petri dishes and incubated in airtight plastic containers under a gas phase of 5% CO_2_, 5% propane, and 5% methane (v/v). The solidified LSM media plates were also placed in airtight jars containing the same headspace gas composition. The plates were incubated statically at 55 °C. Individual whitish colonies that formed on the membrane filters or plates were transferred to fresh liquid media. Isolated strains were designated IT5 and IT6. For comparative studies, a mesophilic methanotrophic strain designated B4 was isolated from cultures enriched in the same media at 42 °C with methane as a sole energy source.

Microscopic observation of wet mounts prepared from the isolated colonies revealed a single rod-shaped cell morphology. To verify that there was no contamination by heterotrophs, aliquots of the cultures were seeded into liquid and phytagel-solidified LSM medium supplemented with 0.25% (w/v) Luria broth and tryptic soy broth at pH 4.5 and incubated without methane or propane at 55 °C. To further verify the purity of the isolated strains, genomic DNA of three independently grown cultures of each strain was extracted with a genomic DNA extraction kit (Exgene Soil DNA mini, GeneAll, Korea) according to the manufacturer’s instructions. DNA of each sample was used as templates for PCR amplification of the bacterial and archaeal 16S rRNA genes with the primers pairs 27F/1492R [50] or 20F/958R [51]. Sanger sequencing of the purified PCR products was performed at Cosmo Genetech, South Korea.

### Growth experiments

Unless otherwise noted, all growth experiments were performed in 160-ml serum vials containing 20 ml of LSM medium adjusted to pH 4.5 with 5 mM MES buffer and inoculated with 1% (v/v) of actively growing cells from the late log phase (starting optical density values at 600 nm (OD_600_) < 0.01). Vials were incubated at 50 °C with shaking at 200 rpm. The growth of isolates was monitored by measuring changes in OD_600_ using a spectrophotometer (Optizen 2120UV, Mecasys Co., Daejeon, Korea).

The utilization of alternative carbon sources other than methane was tested by supplementing LSM medium with alternate carbon compounds (See Table S2 for types and concentrations). The effect of CO_2_ on growth was determined by growing the cells on LSM medium containing methanol (30 mM) and 2-propanol (10 mM) with supplementation of 0–10% (v/v) CO_2_ in the headspace. OD_600_ of the cultures was tracked over time to determine the growth rates of the strains on selected substrates. The growth rates were determined by linear regression of the Log_10_ optical density values versus time.

### DNA extraction and 16S rRNA gene amplicon sequencing

Genomic DNA was extracted from the enrichments and original inocula using an Exgene Soil DNA mini extraction kit (GeneAll, Korea) according to the manufacturer’s instructions. A modified CTAB method [52] was employed in extracting high molecular weight genomic DNA of the isolated strains (strains IT5, IT6, and B4) from 200 ml of culture grown on methane. In brief, biomass obtained from the cultures was treated with CTAB and sodium dodecyl sulfate (SDS) extraction buffer, incubated for 30 min at 65 °C with occasional mixing and centrifuged at 8000 × *g* for 10 min at 25 °C. The supernatant obtained was repeatedly purified with an equal volume of chloroform/isoamyl alcohol (24:1). The extracted DNA was precipitated with 0.6 volume of 2-propanol, and the pelleted DNA was washed twice with 70% ethanol, allowed to air dry, and resuspended in TE buffer (10 mM Tris, pH 8, 1 mM EDTA). The extracted DNA concentrations were measured with a NanoDrop ND-1000 spectrophotometer (Thermo Fisher Scientific, Waltham, MA, United States), and the quality was assessed on a 1% (w/v) agarose gel. For community analyses, 25 ng of the extracted DNA from the enrichments or original inocula were used as templates.

The hypervariable V4-V5 region of the 16S rRNA gene was amplified with the primer pairs 515F/926R [53] and sample indexing adapters (Nextera XT index kit). PCR amplifications were conducted via the following steps: 3 min heating step at 95 °C, followed by 25 cycles at 95 °C for 45 s (denaturation), 50 °C for 45 s (annealing), 72 °C for 90 s (extension), and 72 °C for 5 min (final extension). The PCR product was purified using the Labopass purification kit (Cosmo Genetech, South Korea), and the quantity obtained was measured with a NanoDrop ND-1000 spectrophotometer (Thermo Fisher Scientific, Waltham, MA, United States). The quality of the PCR product was assessed on a 1.5% (w/v) agarose gel. Sequencing of the 300-bp paired-end reads on the MiSeq platform (Illumina) was performed by Macrogen Inc, South Korea. The raw sequence reads were quality controlled with FastQC (ver. 0.11.3) and subject to quality trimming, adapter removal using Trimmomatic (ver. 0.36). Reads pairs were merged with FLASH (ver. 1.2.11) [54], and operational taxonomic units (OTUs) clustering (at a 97% sequence similarity threshold) was performed with CD-HIT-OTU [55]. The clustered OTUs were mapped to the SILVA database (ver. 138) on QIIME2 [56]. Hierarchical clustering with heatmap analysis was performed using the heatmap.2 package in R using Bray-Curtis dissimilarity.

### Genome sequencing, assembly, and annotation

High-throughput sequencing of the genomes was performed at Macrogen (Seoul, Republic of Korea). The sequencing reads were obtained from PacBio RSII and HiSeq (Illumina) according to the manufacturer’s instructions. The PacBio sequencing and HiSeq (Illumina) DNA libraries were prepared with the 20 kb SMRTbell TPK kit and Nextera XT Library Preparation kit, respectively. The error-corrected long reads were assembled using the HGAP assembler (ver. 3.0). The assembled genomes were annotated with the NCBI Prokaryotic Genome Annotation Pipeline [57] and MicroScope annotation platform [58]. To predict gene functions, derived protein sequences were searched using BLASTp (ver. 2.9.0) [59] against the NCBI NR database (updated 20 May 2020). Based on protein sequences, we identified the orthologous genes between strains IT5, IT6, and other verrucomicrobial methanotrophs using the Pan-genomes analysis pipeline (ver. 1.2.1). For this analysis, we employed the default parameters, a minimum alignment coverage of 50% and a minimum alignment identity of 50%, to identify the protein sequences. The complete genome sequences of “Methylacidiphilum sp.” IT5, “Methylacidiphilum sp.” IT6, and “Methylacidimicrobium sp.” B4 were deposited in the NCBI GenBank as accession numbers CP065956, CP065957, and CP066203, respectively.

### Phylogenetic analysis

For phylogenetic analyses, reference 16S rRNA and *pmoA* (encoding methane monooxygenase subunit A) gene sequences of representative strains were obtained from the NCBI NT and NR databases, respectively. The reference sequences and sequences from this study were aligned with CLUSTALW [60] in BioEdit [61], and phylogenetic trees were constructed with the neighbor-joining method and 1000 bootstraps in MEGA7 [62]. Calculation of the 16S rRNA gene similarity was performed using EzBioCloud [63].

### Transcriptomic analysis

Transcriptome experiments were conducted under O_2_-replete conditions (18% v/v O_2_ in the headspace) in 160-ml vials fitted with O_2_ sensors. The 160-ml vials contained 20 ml medium and a headspace containing 5% (v/v) CO_2_ and 18% O_2_. Methane (10%, v/v), 2-propanol (10 mM), acetone (10 mM), and acetol (10 mM) were used as energy sources individually. For the oxygen-replete conditions, cells were harvested before 25% of the initial O_2_ (corresponding to 30% of the initial methane) was consumed. The O_2_ mixing ratio at harvest was therefore still >13% v/v of the total gas headspace. For comparison, O_2_-limited incubations were conducted in 1-L serum vials containing a culture volume of 50 ml supplemented with 10 mM acetone and with 3.5% O_2_ (v/v) in the headspace. For all conditions, the biomass was sampled before the OD_600_ reached 0.15. A 20-ml volume of cells sampled from each condition was harvested at 5000 × *g* (10 min, 25 °C). The pellets formed were immediately resuspended in 4 ml of RNAlater RNA Stabilization Solution for Tissue (Sigma-Aldrich, Seoul, South Korea) and stored at −80 °C until analyses. The total RNA was extracted from the harvested cells in triplicates using the mirVana miRNA Isolation Kit (Thermo Fisher Scientific, Seoul, South Korea) according to the manufacturer’s protocol. The total RNA quality was checked using the Agilent 2100 Expert Bioanalyzer (Agilent), and the cDNA libraries were prepared with the Nugen Universal Prokaryotic RNA-Seq Library Preparation Kit. According to the manufacturer’s instructions, the cDNA libraries were sequenced via NovaSeq6000 sequencing (Illumina) at DNALink (Seoul, South Korea). The raw reads were filtered with FastQC (ver. 0.11.8). To obtain mRNA reads, the reads mapping with rRNA and tRNA genes of strain IT6 were removed using Bowtie2 and SAMtools. The transcripts were mapped against the genes of strain IT6, and the mapped reads to each gene were counted using SAMtools. Expression values are shown as transcripts per kilobase million (TPM). Putative promoter sequences in the target DNA fragment (spanning the gene cluster 9370-9425 regions) were predicted by an online alignment search tool, BPROM [64], and further trimmed using the online software CNNpromoter program [65]. The terminator-finding web software ARNold [66], was also employed to improve the quality of promoter prediction. The statistical analysis of differentially expressed genes was performed using the DESeq2 package in R. UPGMA hierarchical clustering with heatmap analysis was performed with the heatmap.2 package using Bray-Curtis dissimilarity in R. The volcano plot was created in SigmaPlot (ver. 10.0) by plotting the fold-change (FC) values (log_2_FC) against the −log_10_ false discovery rates (FDR). The transcriptomics data of strain IT6 were deposited in the NCBI BioProject database under the accession number PRJNA684446.

### Effect of allylthiourea (ATU) and copper concentration on substrate utilization

The effects of ATU and copper limitation on the growth of strain IT6 were analyzed in the presence of various substrates. Changes in OD_600_ over time were monitored when cultures of strain IT6 were grown in LSM medium containing allylthiourea (0, 25, 50, and 500 µM) as an inhibitor of the CuMMO enzyme [14, 24, 67]. The following growth substrates were used: methane (10% v/v headspace), methanol (30 mM), isopropanol (10 mM), acetone (10 mM), and hydroxyacetone (10 mM). To test the effect of copper concentration on the growth rates of strain IT6, the LSM medium used was supplemented with TES1 without added copper (See Table S1 for details). Copper concentrations of 0 and 10 nM were added to make a copper-deficient and copper-containing LSM medium, respectively. Inocula used were prewashed with a copper-free medium two times by centrifugation at 5000 × *g* for 20 min to avoid trace metals transfer from the culture medium. The solutions used for the experiment were prepared with ultrapure water (Ultra Trace Elemental Analysis Grade; Fisher), and glassware was prewashed twice with 1 M HCl, rinsed three times with Milli-Q-water, autoclaved, and dried at 65 °C before use.

### Resting-cell assays

To estimate oxidation rates of various substrates by the PMOs, the activity of concentrated cells grown on methane and acetone was analyzed. A volume of 200 ml of cells was harvested from batch cultivations grown under the substrate of interest by centrifugation (5,000 × *g* for 20 min) at 25 °C. Harvested cells were washed at least two times with basal LSM medium (pH 4.5) lacking a nitrogen source, trace elements, vitamins, or a carbon source. The washed cells were resuspended in the same medium to achieve varying cell concentrations. To test the possibility that substrate degradation was limited due to mass transfer, methane oxidation was tested in varying concentrations of cells (at OD_600_ = 0.24, 0.48, and 1.0). Three milliliters of cells were transferred to 28-ml vials capped gas-tight with butyl rubber stoppers. Methane (1% v/v) was added to the headspaces to achieve a dissolved methane concentration of 8.6 μM based on Henry’s constant (50 °C, 1 atm) [68]. Mass transfer limitation was not observed as the measured rates were proportional to the cell density used. Thus, activity assays with three milliliters of resting cell suspension at OD_600_ = 1 were conducted using gaseous and liquid substrates. Due to the linear decrease of methane over time at 1% (v/v), this mixing ratio was used to estimate oxidation rates of gaseous substrates. To prevent cell growth, a nitrogen source and trace metals were not provided, but chloramphenicol (1.25 mg/l) was added. Formate (10 mM) and acetol (1 mM) were supplemented to generate reducing equivalents for methane-grown cells and acetone-grown cells, respectively, at pH 4.5. The suspensions were incubated for 2–3 h with the substrate of interest (at 1 mM or 1% v/v) in 28-ml vials at 50 °C with shaking at 300 rpm. The amount of substrate consumed, or product formed over time was detected by gas chromatography as described below.

To complement the limitations of the gas chromatograph-based resting cell activity assay, comparative kinetics of methane and acetone oxidation were estimated by measuring substrate-dependent O_2_ consumption rates over time. For this purpose, a 2-ml respiration chamber fitted with a contactless oxygen sensor spot (OXSP5, PyroScience, Germany) was filled with whole-cell suspensions of strain IT6 (at OD_600_ = 0.2) prepared as described above. The oxygen signal in the closed chamber was monitored on a FireSting fiber-optical oxygen meter (FSO2-1, PyroScience, Germany) for more than 10 min to confirm the initial equilibration before substrate injection. Equipment operation and two‐point calibration were done in line with the manufacturer’s instructions. The chamber was operated at 50 °C (Δ*T* < 0.1 °C) with a reciprocating shaking water bath (NB-304, N-BIOTEK Co., South Korea) and stirred with a magnetic stirrer (MIXdrive 1 XS, 2mag AG, Germany) at 1000-rpm. The amount of methane and acetone added was within the range of 0.5–200 µmol, and the substrate-induced oxygen consumption rate was calculated considering the endogenous respiration rate. A saturated-aqueous methane solution was prepared in a 160-ml serum bottle filled with 100 ml of LSM medium and flushed with 100% (v/v) methane. Total methane and acetone oxidation rates were determined from microsensor measurements of substrate-dependent O_2_ consumption in single-trace measurements. The stoichiometry of methane to oxygen consumption was estimated at 1:1.55, while acetone to oxygen consumption was estimated at 4:1. The kinetics constants, *K*_*m*(app)_ and *V*_*max*_ were calculated by fitting a Michaelis–Menten equation (Eq. 1) to O_2_ and substrate consumption rates, while the specific affinity (*a*^*o*^) was estimated from the *K*_*m*(app)_ and *V*_*max*_ values (Eq. 2) as described previously [69].

1 $$V = (V_{max} \times \left[ {\mathrm{S}} \right]) \times (K_{m(app)} + \left[ {\mathrm{S}} \right])^{ - 1}$$

2 $$a^{\mathrm{o}} = V_{max} \times K_{m(app)}^{ - 1}$$

where *V* denotes the oxidation rate (expressed in mmol h^−1^ mg-dry cells^−1^), *V*_*max*_ is the maximum rate (expressed in mM h^−1^), *K*_*m*(app)_ is the apparent Michaelis–Menten half-saturation constant (in µM), [*S*] the substrate concentration (in µM), and *a*^*o*^ is the specific affinity (L g dry cells^−1^ h^−1^).

### Analytical methods

The mixing ratios of gas substrates in the headspaces were monitored using a gas chromatograph (GC-2010 Plus, SHIMADZU, Japan) equipped with an Rtx1- capillary column (30 m × 0.25 mm × 0.25 µm, Restek, Bellefonte, PA, United States) coupled to a flame ionization detector. The carrier gas was dinitrogen, and the following conditions were employed: injector temperature of 150 °C with a split ratio of 1:10, oven temperature of 80 °C, and detector temperature of 200 °C. A calibration curve of the gases used as substrates was prepared from pure gases.

Quantification of liquid substrates was performed via GC using a DB-WAX column (30 m × 0.25 mm × 0.25 µm; Agilent Technologies Inc., USA). The carrier gas used was dinitrogen and the following conditions were employed; injector temperature of 200 °C with a split ratio of 1:6, the oven temperature was 90 °C for 3 min then increased at a gradient of 10 °C/min to 220 °C and held for 5 min. The quantification of 2-propanol, acetone and acetol was done by comparison with standard aqueous solutions. To measure liquid substrate consumption by the cells, aliquots (200 µl) of the cultures were sampled over time and centrifuged at 15,000 × *g* for 10 min at 25 °C room to pellet the cells. The supernatant was removed and used for substrate concentration determination. An aliquot of the supernatant (1 µl) was injected into the GC.

For dry weight determination, cells were harvested from batch cultures by centrifugation (5000 × *g* for 20 min) at 25 °C. The harvested cells were washed two times with LSM medium and adjusted to an optical density of 0.5 (OD_600_) with liquid medium. Samples of five milliliters of the cell suspension were filtered through pre-weighed 0.2-µm polycarbonate filters (Whatman, UK) and dried in a vacuum oven at 60 °C until a constant weight was achieved. The same volume of LSM medium was filtered through membranes to calculate the blank.

## Results and discussion

### Enrichment and isolation

To investigate the oxidation of gaseous hydrocarbons in the Pisciarelli hot springs samples, the following energy sources were added to the headspaces over a minimal medium: (1) methane, (2) propane, (3) propane+methane. There was no propane-oxidizing activity in the enrichments containing propane as the sole energy source. In the enrichments with propane+methane, propane oxidation was observed only in the cultures that displayed active methane oxidation (Fig. S1). Furthermore, the onset of propane oxidation was delayed, becoming detectable only a few days after the onset of methane oxidation. With the consumption of only 5% v/v methane, oxygen is assumed to still be replete for propane oxidation. Methane oxidation was delayed when propane was present compared to when only methane was present. When these enrichment cultures were transferred to fresh medium with propane as the sole energy source, propane was no longer oxidized. These results all suggest a cometabolic oxidation of propane by methanotrophs.

**Fig. 1:**
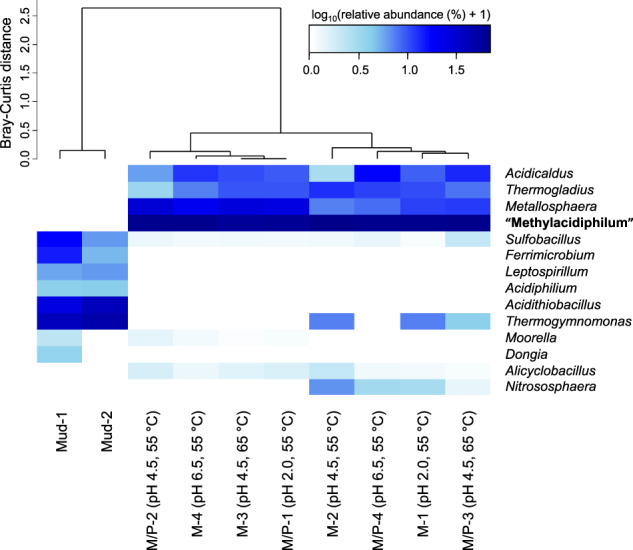
Cluster analysis of the microbial community in Italian volcanic mud samples and enrichment cultures. Compositional dissimilarities between samples were quantified using Bray-Curtis dissimilarity. The community composition was obtained by 16S rRNA gene amplicon sequencing analysis. ‘M’ denotes cultures with methane. ‘M/P’ denotes cultures with methane plus propane. ‘Mud’ denotes the original sample. The color scale (from white to blue) represents the natural logarithm transformed values of the percentage relative abundances +1. Genera with individual abundances less than 1% together comprised <3% of the total community and were excluded from the analysis.

Analysis of microbial communities using 16S rRNA gene amplicon sequencing showed that communities grown under methane and propane+methane were similar to each other but distinct from those of the original samples (Fig. 1). OTUs specific to propane+methane-consuming enrichments and belonging to taxa known to oxidize propane could not be identified. An OTU belonging to the genus “Methylacidiphilum” of the phylum Verrucomicrobia was found to be prominent in the methane (60−66% relative abundance) and propane+methane (62−65% relative abundance) enrichment cultures (Fig. S2). Recently, the verrucomicrobial methanotroph, “M. fumariolicum” SolV, cultivated on natural gas, oxidized ethane and methane simultaneously and also oxidized propane after methane and ethane became limiting [8]. In our enrichment, the delay of methane oxidation in the presence of propane might be attributable to competitive inhibition by propane. It was previously observed that ethane competitively inhibits the growth of strain SolV [13], and propane is likely to have a similar effect. Thus, the dominance of “Methylacidiphilum”, previously thought to be obligate methanotrophs or hydrogenotrophs [30, 70, 71], in our methane+propane cultures, ties well with this recent finding.

**Fig. 2:**
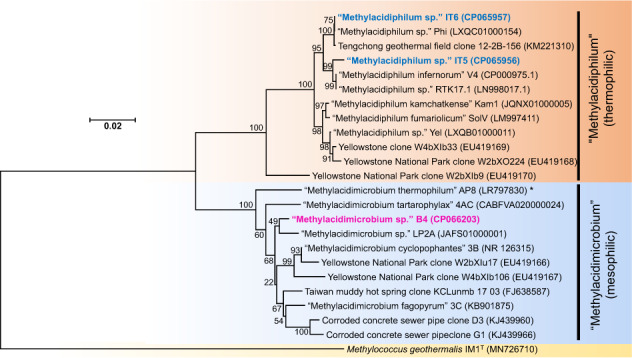
Phylogenetic positions of isolated strains in relation to other verrucomicrobial methanotrophs based on 16S rRNA gene sequences. The tree was constructed with MEGA 7 using the Neighbor-joining method. The evolutionary distances were computed using the Kimura 2-parameter method and are in units of base substitutions per site. The rate variation among sites was modeled with a gamma distribution (shape parameter = 5). All positions with less than 95% site coverage were removed, leaving a total of 1461 positions in the final dataset. The gammaproteobacterial methanotroph *Methylococcus geothermalis* was used as the outgroup. ‘*’ indicates that “Methylacidimicrobium thermophilum” is the only known thermophilic strain of the genus, “Methylacidimicrobium”. The scale bar represents 0.02 changes per nucleotide position.

After successive transfers of the cultures with the greatest propane-oxidizing activity (at pH 4.5 and 6.5 and 55 °C) (Fig. S1) to fresh media, colonies growing under the same gas atmosphere were isolated using a floating filter technique. Morphologies of the isolates (Fig. S3) were similar to those of verrucomicrobial methanotrophs [9, 11, 36]. Based on phylogenetic analysis of 16S rRNA gene sequences, isolates IT5 and IT6 were classified as thermoacidophilic verrucomicrobial methanotrophs of the proposed genus “Methylacidiphilum”, showing 99.38% and 98.96% similarity, respectively, with “Methylacidiphilum infernorum” strain V4 (Fig. 2). In addition, strain IT6 shared 100% similarity with “Methylacidiphilum” sp. Phi, isolated recently from an acidic hot spring in the Philippines [36].

### Substrate range

**Fig. 3:**
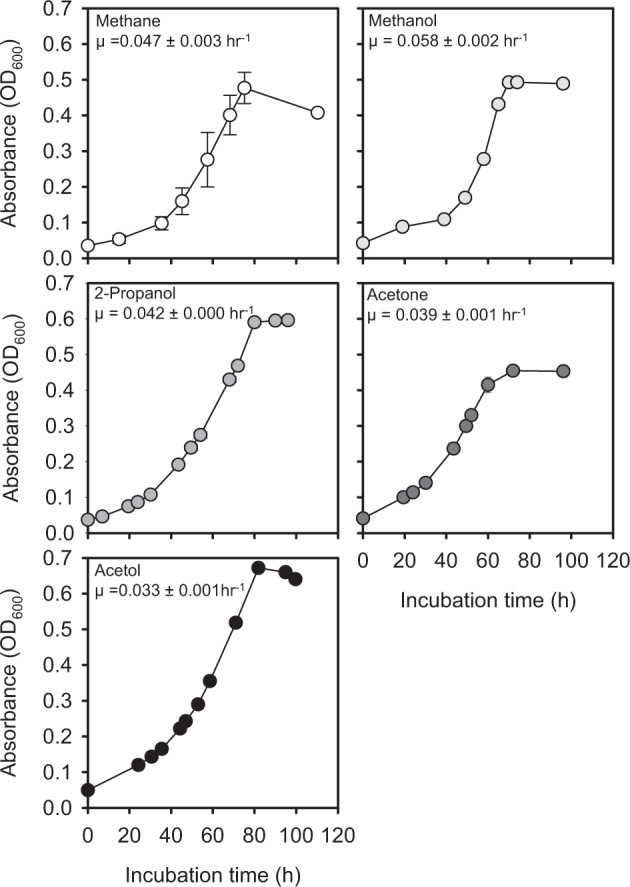
The growth of strain IT6 on C1 and C3 substrates. The cultivation was performed at pH 4.5 and a temperature of 50 °C with shaking at 200 rpm. The error bars represent ±1 standard deviation for *n* ≥ 2 biological replicates. For inoculation, 10% (v/v) of late log phase cells (starting optical density values at 600 nm (OD_600_) < 0.05) were used. Substrates were: 10% methane (v/v), 30 mM methanol, and 10 mM each of 2-propanol, acetone, or acetol.

To test possible utilization of gaseous hydrocarbons by strains IT5 and IT6, the growth substrate range of the isolates was examined and compared with that of “M. infernorum” strain V4 and a mesophilic “Methylacidimicrobium” isolate, strain B4 (Table S2). Strains IT5 and IT6 were able to grow on methane, methanol, and formate (at pH 4.5, which is above formate pKa, 3.8) [41] as energy sources, and their growth was highly dependent on CO_2_ supplementation (Fig. S4A), as previously observed in other “Methylacidiphilum” strains [9, 13, 41, 72, 73]. Propane or ethane (2 or 20%, v/v) alone did not support growth in either oxygen-replete or oxygen-limiting conditions, as expected for obligate methanotrophs [74]. However, strains IT5 and IT6 showed an unexpected capability to grow solely on several C3 compounds in the absence of methane: 2-propanol, acetone, acetol (1-hydroxyacetone), and propane-1,2-diol (Table S2). Growth rates of strain IT6 on methane, methanol, and C3 substrates were comparable (0.033−0.058 h^−1^) (Fig. 3). Due to the similarity of the two thermophilic isolates in their substrate range, further characterization focused on strain IT6 alone. In contrast to growth on methane, growth on the C3 substrates was not dependent on CO_2_ supplementation (Fig. S4B), indicating carbon assimilation from C3 substrates. Notably, the C3 substrates utilized by strain IT6 are typical intermediates of canonical propane oxidation pathways [75, 76].

The growth of these strains on C3 substrates was rather surprising since growth of previously isolated verrucomicrobial methanotrophs was not supported by multi-carbon compounds [9, 12]. In fact, growth on C3 substrates has not been observed in any PMO-utilizing methanotroph. Methanotrophs of the genus *Methylocella*, which use only soluble di-iron monooxygenases for methane oxidation, grow on diverse substrates including the C3 compounds pyruvate, propane, propanol, acetone, acetol, methyl acetate, and propanediol [77]. However, methanotrophs that use PMO enzymes for methane oxidation are much more limited in their substrate ranges. Although utilization of acetate and ethanol has been reported in some alphaproteobacterial methanotrophs with PMOs, i.e., some *Methylocapsa* and *Methylocystis* spp. [78, 79], none have previously been shown to grow on C3 compounds.

### Genomic properties

Intrigued by the utilization of intermediates of propane oxidation, we investigated the mechanism behind C3 substrate utilization through combined genomic and transcriptomic analyses. Genome sequences of strains IT5 and IT6 were analyzed and compared with those of other “Methylacidiphilum” strains. The general genomic features and key genes predicted for C1 metabolism are described in Tables S3 and S4, respectively. Based on the genomic analysis, the methane oxidation and carbon assimilating machineries of the isolates appear to be very similar to those described in other verrucomicrobial methanotrophs [33–35]. Strains IT5 and IT6 both possess three complete *pmoCAB* operons that are phylogenetically related to those of other verrucomicrobial PMOs (Fig. S5), plus an orphan *pmoC* copy (Table S4). We did not identify genes encoding diiron monooxygenases in any strain. Compared to the genome of “M. infernorum” strain V4, there were 132 and 208 ORFs (7% and 10% of the total ORFs of the genomes, respectively) unique to strains IT5 and IT6, respectively. Among them, the number of shared ORFs between the genomes of strains IT5 and IT6 was only 17, and predicted functions of the ORFs were not related to the metabolism of the C3 substrates (Table S5). Thus, a comparative genomic analysis failed to identify genes encoding enzymes for C3 substrate oxidation that were exclusive to strains IT5 and IT6.

### Transcriptomic analysis

**Fig. 4:**
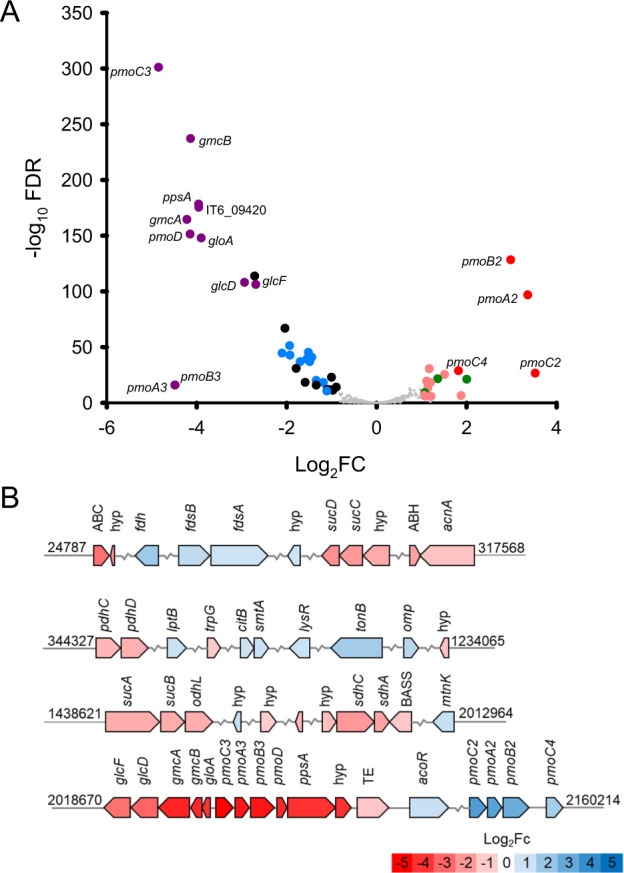
Comparative gene expression pattern of strain IT6 grown on C1 and C3 substrates. **A** A volcano plot showing the differential gene expression between 2-propanol-grown (left) versus methane-grown cells (right) under oxygen-replete conditions. The volcano plot was generated with the fold-change values (log_2_FC) and false discovery rates (FDR) from three replicates of both conditions, with the methane grown-cells used as the reference condition. Genes with less than two-fold expression difference (FC ≤ 2 and FDR ≥ 0.05) are represented by small gray dots and other genes of interest with more than two-fold expression difference (FC ≥ 2 and FDR < 0.05) in 2-propanol and methane-grown cells are represented by large colored dots with the following description: (1) Purple, blue and black are genes in cluster IT6_09370-09425, TCA cycle genes, and other upregulated genes, respectively, in 2-propanol-grown cells, (2) Red, green and pink are genes involved in methane oxidation, formate oxidation, and other processes, respectively, in methane-grown cells. Genes in purple (cluster IT6_09370-09425) and red (Genes for methane oxidation) are labeled. **B** Organization of genes with more than two-fold expression difference (FC ≥ 2 and FDR < 0.05) in 2-propanol-grown and methane-grown cells. The genes colored in blue were expressed relatively more during growth on methane, while genes colored red were expressed relatively more during growth on 2-propanol. The color intensity indicates the relative fold change difference (log_2_FC). The numbers at the beginning and end of the genomic region indicate the nucleotide positions of the genes in the strain IT6 genome. The zigzag line indicates more than 1.5 kb distance between two genes. Arrows show gene direction and relative size. Detailed information on the genes can be found in Supplementary Table S7.

**Fig. 5:**
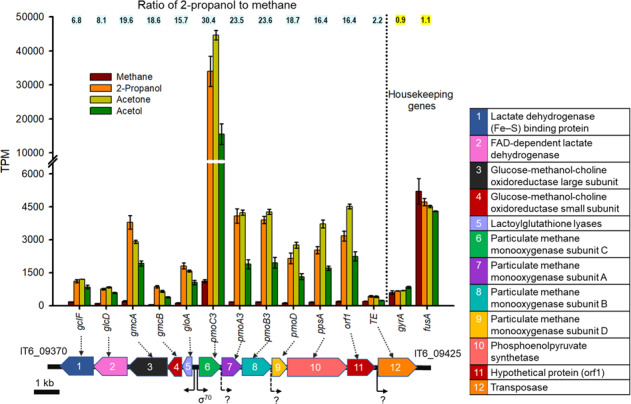
Expression and promoter analyses of the genes localized in the cluster 9370-9425 in strain IT6. Each bar represents gene expression levels (in transcript per million reads, TPM) for cells grown on methane, 2-propanol, acetone, and acetol, as indicated by the color bars. Expression of the housekeeping genes *gyrA* and *fusA* are shown for comparison. Error bars represent ±1 standard deviation for *n* ≥ 3 biological replicates. Apart from the housekeeping genes, differences in expression are statistically significant for each gene (*p* < 0.05). The gene cluster arrangements are shown and represented by different colors (except for genes with hypothetical functions shown in red). Black arrows below the cluster represent the promoter prediction results (LDF score of 0.2) in the gene cluster. An LDF score of 0.2 indicates the presence of an ±RpoD (σ70) promoter with 80% accuracy and specificity.

To identify genes involved in C3 substrate oxidation, we performed a genome-wide transcriptomic analysis of strain IT6 grown on various C3 substrates (2-propanol, acetone, or acetol) as sole energy sources and compared them to cultures grown on methane. Our transcriptomic data could be assumed to be robust since high correlation coefficient values (>0.90) were obtained when logarithmic values of TPM+1 of 384 housekeeping genes at each condition (in triplicates) were plotted against each other (Fig. S6 and Table S6). When strain IT6 was grown on different C3 substrates, the expression profiles were similar to each other but distinct from the cells grown on methane (Fig. S7A). Between 32 and 52 genes were significantly upregulated (FC ≥ 2 and FDR < 0.05) on 2-propanol and other C3 substrates (Fig. 4, Fig. S7B, C, and Table S7). Interestingly, the top 12 most highly expressed genes (>4-fold) were localized in a gene cluster (IT6_09370−09425) (Fig. 5). Surprisingly, this cluster included the *pmoCAB3* operon encoding the most divergent CuMMO (“PMO3”), which was highly upregulated (14- to 40-fold) in C3 substrate-grown cells. In contrast, the operon encoding PMO2 was highly upregulated (7- to 11-fold) only in methane-grown cells (Fig. 4, Fig. S8, and Table S4). The *pmoCAB1* operon, which has been previously demonstrated to be highly expressed under O_2_-limiting condition [38, 40] and more recently under oxygen-replete conditions during growth on methanol+SCA [8], was barely expressed in any of the growth conditions tested in strain IT6 (Fig. S8). The genes and their synteny in the upregulated cluster (IT6_09370−09420) were highly conserved in the genomes of previously studied thermophilic verrucomicrobial methanotrophs (Fig. S9 and Table S8). We also predicted promoter sequences within this cluster likely involved in the transcriptional regulation. Theoretical prediction of promoters revealed that three sets of genes (*gclF* to *gloA*, *pmoA3* to *pmoB3*, *pmoD* to orf1) are expected to be coregulated as operons at the transcriptional level (Fig. 5). Independent regulation was only predicted in the transcription of *pmoC3*. Interestingly, a comparable structural organization of gene clusters spanning *pmoC* to *pmoD* was previously reported [80, 81]. Further experimental analyses in the transcription regulation could provide additional insight into *pmoCAB3* and related gene regulation at the transcriptional level. Apart from the clustered genes, a further 11 of the 32 total C3-upregulated genes encoded for TCA cycle enzymes (Table S7), supporting heterotrophic growth on the C3 substrates. Unexpectedly, genes encoding the large and small subunits of RuBisCO (*cbbL* and *cbbS*, respectively) for the Calvin–Benson–Bassham cycle were constitutively expressed in all conditions (Table S4), indicating that CO_2_ might be the principal carbon source of verrucomicrobial methanotrophs in acidic geothermal environments.

### C3 substrates metabolic pathway

To predict the utilization pathway for C3 substrates, the highly expressed gene cluster was analyzed in detail. Genes in the cluster (Fig. 5) were predicted as follows: (i) lactate dehydrogenase (IT6_09370 and 09375), *(*ii*)* glucose-methanol-choline (GMC) oxidoreductase (IT6_09380 and IT6_09385), (iii) lactoylglutathione lyases (IT6_09390), (iv) *pmoCAB3* operon (IT6_09395–09405) with an additional *pmoD* gene (IT6_09410), (v) phosphoenolpyruvate (PEP) synthase (IT6_09415), (vi) unknown (IT6_09420), and (vii) transposase (IT6_09425).

Oxidation of 2-propanol to acetone is expected to be initiated by an alcohol dehydrogenase. Although *xoxF* was highly expressed (TPM value > 3100) on all substrates used in this study (Table S4 and Fig. S10), the XoxF of SolV, which is highly similar to that of strain IT6, had no activity toward 2-propanol [82]. Interestingly, GMC oxidoreductase genes were highly upregulated in 2-propanol-grown cells (19- to 20-fold). The GMC family oxidoreductases are versatile and can carry out alcohol oxidation reactions in the cytoplasmic membrane facing the periplasm with a consensus motif of twin-arginine-dependent translocation signal peptide [83, 84]. Thus, the GMC oxidoreductase may function as 2-propanol dehydrogenase in the periplasm.

Microbial acetone oxidation can proceed via either a CO_2_-dependent or an O_2_-dependent mechanism [85–88]. The CO_2_-dependent pathway involves acetone condensation with CO_2_ to yield acetoacetate by an ATP-dependent acetone carboxylase (AcxABC) [85]. On the other hand, the O_2_-dependent mechanism can proceed via two possible pathways: (1) insertion of an oxygen atom into the C-C bond of acetone by FAD-dependent monooxygenase (encoded by AcmA) to yield methyl acetate [86] and (2) hydroxylation of the methyl group in acetone to yield acetol by a cytochrome P450 monooxygenase [88] or a diiron monooxygenase [87]. Despite its growth on acetone, no genes encoding canonical acetone oxidation could be identified in strains IT5 and IT6, suggesting that acetone oxidation proceeds via a novel mechanism. High upregulation of the *pmoCAB3* operon encoding PMO3 in the C3 substrate-grown cells and its close proximity to other genes implicated in the downstream oxidation of acetol (see below) is intriguing. Within the last two decades, highly divergent copies of the *pmo* operon, encoding CuMMO with unknown substrate specificities, have been discovered in the proteobacterial [16, 28, 29] and verrucomicrobial methanotrophs [9, 13, 30] as well as in non-methanotrophic heterotrophs [15, 42, 43]. Some of these new CuMMOs have been implicated in the oxidation of non-methane hydrocarbons [14, 15], but none have been proposed to target short-chain ketones specifically. Although *pmoCAB3* expression was increased in a SolV culture grown on methanol+propane, its role in propane degradation could not be resolved since the *pmoCAB1* was primarily implicated in propane oxidation [8]. Our results provide a strong evidence for the involvement of the verrucomicrobial PMO3 in an acetone oxidation reaction analogous to cytochrome P450 monooxygenase or a diiron monooxygenase.

**Fig. 6:**
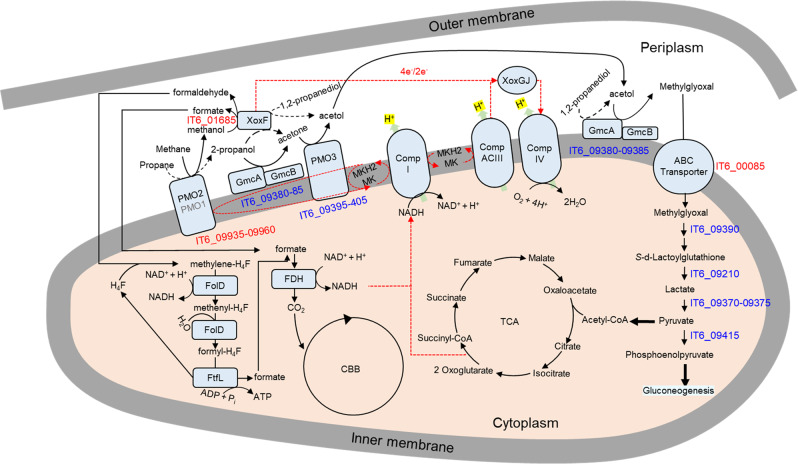
The proposed metabolic pathway for C3 substrate oxidation in strain IT6. The methane monooxygenases (PMO1 and PMO2) are involved in the oxidation of methane and other SCA [8] to their respective alcohols. The lanthanide-dependent methanol dehydrogenase (XoxF) is involved in the conversion of methanol to formaldehyde or formate. The XoxF or other alcohol dehydrogenases (e.g., the GMC oxidoreductase complex transported to the periplasm via the TAT system; GMC large subunit (GmcA) and small subunit (GmcB)) encoded by the strain might be involved in converting 2-propanol and 1,2-propanediol to acetone and acetol, respectively. The reduced quinones can provide the reducing equivalents for PMO2 and PMO3 from methanol oxidation and 2-propanol oxidation pathways. Acetone generated from 2-propanol is oxidized to acetol through the action of the PMO3. The acetol produced is oxidized to methylglyoxal by GMC oxidoreductase. Transport of methylglyoxal into the cytoplasm can occur via passive diffusion or possibly via a transporter. The glyoxalase (I and II) enzyme system convert methylglyoxal to lactate, further converted to pyruvate by lactate dehydrogenase. The pyruvate generated can be converted to PEP by PEP-synthase, an essential step in gluconeogenesis during growth on the C3 substrates. The alternative complex III (Comp ACIII) transfers electrons from reduced quinones via a cytochrome *c* (Cyt-*c*) to complex IV, thereby regenerating the quinone pool. The respiratory complexes translocate protons across the cellular membrane. Pyruvate enters the TCA cycle for ATP/NADH production and anabolic processes. For selected predicted reactions for C1 and C3 metabolism, the gene identifiers are shown in blue for genes in cluster IT6_09370-09425 and red for others. Black dashed arrows represent reactions for which the candidate enzyme was not confidently predicted.

A previous proteomics study revealed that the GMC oxidoreductase was not expressed in methane- or succinate-grown cells of *M. silvestris* BL2 but was strongly expressed in cultures amended with propane [89]. A recent study revealed that the GMC oxidoreductase of *M. silvestris* BL2 was responsible for the oxidation of acetol to methylglyoxal (2-oxopropanal) [27]. Thus, GMC oxidoreductase (IT6_09380 and IT6_09385) would allow the conversion of acetol to methylglyoxal as well as 2-propanol to acetone (see above). The reaction converting methylglyoxal to lactate consists of two steps. Lactoylglutathione lyase (glyoxalase I) (IT6_09390) may catalyze the first reaction step [90, 91]. There were six candidate genes of Zn-dependent hydrolase (glyoxalase II) (Table S9) for the second step reaction in the IT6 genome, but these were located distantly from the C3-upregulated cluster, and their expression was not differential. The two upregulated genes (IT6_09370 and 09375) containing Fe-S- and FAD-binding motifs, respectively, comprise an annotated lactate dehydrogenase [92]. PEP synthetase (IT6_09415) is required for the synthesis of precursor metabolites for cellular carbon compounds via gluconeogenesis [93]. Based on the genomic and transcriptomic analysis results, we propose a metabolic pathway of C3 substrate utilization, as shown in Fig. 6.

### PMO3 as acetone monooxygenase

**Fig. 7:**
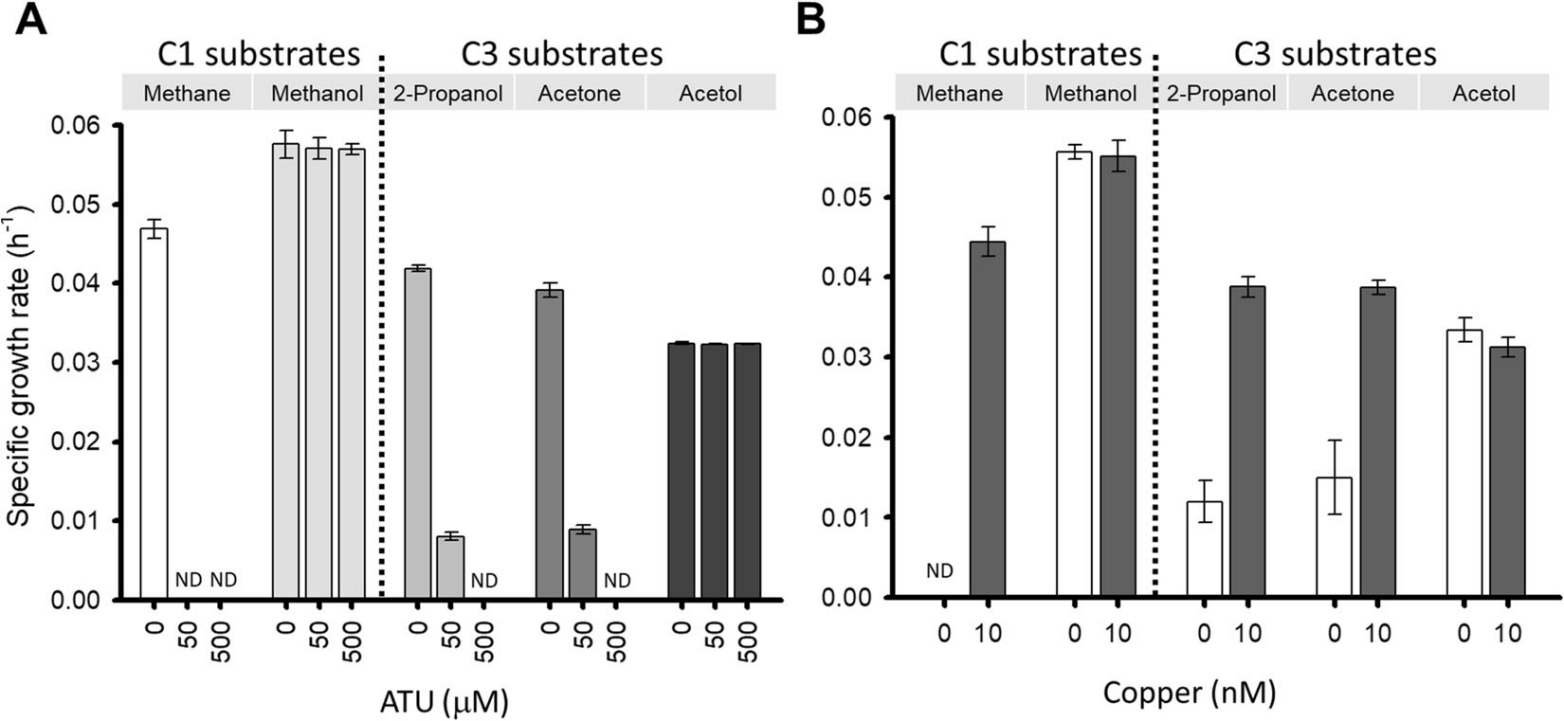
Effect of ATU and copper concentration on the growth of strain IT6. **A** shows the specific growth rate of strain IT6 under varying concentrations of allylthiourea when grown on C1 and C3 substrates. **B** shows the specific growth rate of strain IT6 in the presence and absence of copper (10 nM). Error bars represent ±1 standard deviation for *n* ≥ 3 biological replicates. ND indicates no detectable growth.

Ammonia, methane, and short-chain alkanes are the only natural substrates of the CuMMOs so far documented [14, 17, 18]. The idea that a CuMMO could hydroxylate the methyl group in acetone to yield acetol is fascinating and identifying such a process would expand the known physiological roles of CuMMOs. To support this idea, the effect of a mechanistic inhibitor of CuMMOs, the copper-chelating compound allylthiourea (ATU) [14, 24, 67], on the growth of strain IT6 was tested. Microorganisms that utilize CuMMO are strictly dependent on copper for enzyme expression and activity due to the presence of copper centers in active sites of the enzyme [24, 94]. From a preliminary experiment, it was found that concentrations ≥50 µM ATU were enough to completely inhibit methane oxidation in strain IT6 (Fig. S11). Then, concentrations of 50 and 500 µM ATU were applied to cultures containing methane, methanol, isopropanol, acetone, or acetol as a sole energy source. As expected, growth of strain IT6 on methane, 2-propanol and acetone was strongly inhibited by ATU, whereas growth on methanol and acetol remained unchanged regardless of ATU (Fig. 7A and Fig. S11). Similar results were obtained when the growth of strain IT6 was compared in copper-free media versus media supplemented with 10 nM copper (Fig. 7B). Together, a crucial role for copper during growth on methane and acetone (and 2-propanol) is most likely related to the activity of PMO2 and PMO3, respectively, considering their substrate-specific expression (Fig. S8 and Table S4). Upregulation of *pmoCAB3* under both oxygen-replete and -limiting conditions in acetone-grown cells (Fig. S8) supports its involvement in acetone oxidation. Neither an operon encoding a *pmoCAB3*-like CuMMO nor any other genes encoding C3 substrate utilization (Fig. S9) were found in the mesophilic isolate genome, strain B4, which supports the conclusion that this strain is an obligate methylotroph (Table S2).

### Substrate specificity of PMO3

Although the active site of the CuMMOs is still under intense investigation [94–97], potential mononuclear copper sites (Cu_B_ and Cu_C_) were recently proposed in the B and C subunits, respectively [95]. Evidence of methane oxidation activity by the soluble portion of the B-subunit has been demonstrated, albeit with low activity [94]. The idea that the C-subunit is the active site for substrate binding and oxidation was supported by recent evidence [95–97], and the absence of the three histidine ligands for Cu_B_ in the verrucomicrobial PMOs has been noted [30]. Substrate preference of the hydrocarbon monooxygenase (HMO), a CuMMO enzyme acting primarily on short-chain alkanes, was suggested to be also linked to an amino acid residue in the C-subunit, Ala 151 (site HmoC151) (Fig. S12) [97]. Accordingly, physiologically diverse CuMMO-containing microorganisms were found to have characteristic amino acids (aspartate, glutamate, serine, alanine, or proline) in the corresponding site. While alanine is highly conserved in the site of HMO, aspartate was found to be preferred for this site in PMOs of the methane oxidizers in the Proteobacteria, NC10, and Verrucomicrobia [97]. Interestingly, among the three CuMMOs in “Methylacidiphilum”, glutamate in this key site is observed only in PMO3, suggesting that it may have a unique substrate specificity (Fig. S12).

Resting cell experiments were conducted to compare substrate specificities of PMO2 and PMO3, which were exclusively upregulated on methane and C3 substrates, respectively. Methane-grown cells could not oxidize acetone and vice versa, acetone-grown cells could not oxidize methane (Table 1 and Fig. S13), demonstrating the substrate specificity of these PMOs. Stoichiometric conversion of 2-propanol to acetone by ATU-treated acetone-grown resting cells further supports the role PMO3 as an AcMO (Fig. S14). Acetone-grown cells could oxidize butanone, indicating the specificity of the PMO3 to short-chain ketones. The inability of strains IT5 and IT6 to grow on ethane (Table S2) despite its cometabolic oxidation by both methane- and acetone-grown cells (Table 1), indicates that there is weak, or no activity of the subsequent biochemical steps required for ethanol utilization (Table S2). Ethanol conversion to acetaldehyde can be catalyzed by XoxF, which was highly expressed during growth on all substrates in strain IT6 and was demonstrated to oxidize ethanol efficiently in strain SolV [8]. However, acetaldehyde might not be efficiently utilized by the following pathway partly due to the absence of canonical formaldehyde dehydrogenase. Propane oxidation activity could be observed only in methane-grown cells, albeit at a slower rate compared to methane (Table 1), indicating gratuitous cometabolic oxidation of propane by the normal methane monooxygenase (PMO1 or PMO2) as observed previously in other methanotrophs [98, 99]. Since PMO1 of SolV was suggested to be the key enzyme for SCA oxidation in cells cultivated on methanol+SCA [8], PMO1 or PMO2 could both be involved in SCA oxidation depending on growth conditions. Thus, propane oxidation cannot be maintained in strain IT6 when methane is depleted, and PMO1 or PMO2 is no longer induced. The lack of PMO3 activity toward propane was evident, as propane was not utilized in the presence of C3 substrates alone. Similarly, *pmoCAB3* upregulated in “M. fumariolicum” SolV grown on a methanol+propane mixture could encode the assimilation of C3 intermediates produced by PMO1 [8]. Respirometry-based kinetic analysis showed that affinity of acetone-grown cells to acetone (*K*_*m*(app)_ = 0.27 ± 0.01 µM) (Fig. S15A) was much higher than that of methane-grown cells to methane (*K*_*m*(app)_ = 8.87 ± 0.04 µM) (Fig. S15B), suggesting efficient utilization of cometabolic oxidation products of propane.

**Table 1:** The specific activity of resting cell suspensions of strain IT6 on gaseous alkanes and ketones.

Substrate	Specific activity (mmol h^−1^ g-DCW^−1^)	
	Methane grown cells	Acetone grown cells
Methane	4.03 ± 0.04	nd
Acetone	nd	0.76 ± 0.01
Butanone	nd	0.19 ± 0.01
Ethane	0.75 ± 0.08	0.43 ± 0.03
Propane	0.22 ± 0.01	nd

Data are expressed as means of three biological replicates ±1 standard deviation. nd, not detected. Values (µmol substrate per dry cell weight of strain IT6 per hour) are calculated based on the amount of substrate consumed by resting cell suspension at OD_600_ = 1 incubated in a 30-ml sealed serum vial with 1 mM liquid substrate (acetone, butanone) or 1% (v/v) gas substrate (methane, ethane, propane) injected into the headspace.

In summary, these data suggest that propane is used as a supplemental energy source during growth on methane. This is reasonable, as thermogenic propane does not occur alone but rather as a component of a much larger methane pool [45, 100]. The presence of PMO3 would allow “Methylacidiphilum spp.” to take energetic advantage of the fortuitous cometabolic oxidation of propane that occurs while they are consuming the larger methane pool in these habitats.

## Conclusions

Verrucomicrobial methanotrophs are widely known as the key methanotrophs in acidic geothermal environments. Here, we showed that verrucomicrobial methanotrophs are also involved in the oxidation of the SCAs ethane and propane. Propane was oxidized only in the presence of methane via cometabolic oxidation by methane monooxygenases (PMO1 and/or PMO2). Surprisingly, oxygenated C3 intermediates of propane oxidation pathways fully supported the growth of verrucomicrobial methanotrophs. The complete biochemical pathway for utilizing C3 substrates was predicted by genomic and transcriptomic analyses and supported by physiology and substrate specificity tests. We propose PMO3 as an AcMO with substrate specificity to short-chain ketones, the fourth known class of natural substrates for CuMMOs. These findings shed new light on the metabolic flexibility of verrucomicrobial methanotrophs as C3 substrate-utilizing facultative heterotrophs [37]. Verrucomicrobial methanotrophs are suggested to be key players involved in the oxidation of gaseous hydrocarbons in geothermal environments, although a complete understanding of the metabolic processes behind gaseous hydrocarbon oxidation and the ecological implications of these processes requires more investigation.

## Accession codes

The complete genome sequences of “Methylacidiphilum sp.” IT5, “Methylacidiphilum sp.” IT6, and “Methylacidimicrobium sp.” B4 were deposited in the NCBI GenBank as accession numbers CP065956, CP065957, and CP066203, respectively. The Miseq (Illumina) data of the 16S rRNA gene were deposited in the NCBI Sequence Read Archive (SRA) under accession numbers SRR13262668 - SRR13262677. All the cDNA reads of strain IT6 are deposited under accession numbers SRR13241162 - 13241173 and SRR14267632 - 14267634.

## Supplementary information

Supplementary information

Supplementary excel tables
