# Supplementary material for: Verrucomicrobial methanotrophs grow on diverse C3 compounds and use a homolog of particulate methane monooxygenase to oxidize acetone
Source: ISME J. 2021 Jun 22;15(12):3636–47. doi: 10.1038/s41396-021-01037-2 (PMC8630023; doi:10.1038/s41396-021-01037-2)
Supplement: Supplementary file 1 — Supplementary information [file 41396_2021_1037_MOESM1_ESM.docx]

**Supplementary information**

**This document includes:**

Supplementary Tables

Supplementary Figures

Supplementary References

**Supplementary Tables**

**Table S1.** Composition of trace elements and vitamin solutions used in this study.

| **Composition** | **Stock concentration (mg/l)** | |
| --- | --- | --- |
| **Vitamin solution** | **Vitamin solution** |  |
| D-biotin | 0.02 |  |
| Folic acid | 0.02 |  |
| Pyridoxine hydrochloride | 0.1 |  |
| Thiamin hydrochloride | 0.05 |  |
| Riboflavin | 0.05 |  |
| Nicotinic acid | 0.05 |  |
| DL-calcium pantothenate | 0.05 |  |
| Vitamin B12 | 0.01 |  |
| P-aminobenzoic acid | 0.05 |  |
| Lipoic acid (thiotic acid) | 0.05 |  |
| 1,4-naphthaquinone | 0.04 |  |
| Nicotinamide | 0.1 |  |
| Hemin | 0.01 |  |
| **Trace element solution 1 (TES)** | **TES1 with Cu^2+^** | **TES1 without Cu^2+^** |
| CuCl_2_ × 2H_2_O | 2 | - |
| MnCl_2_ × 4H_2_O | 100 | 100 |
| H_3_BO_3_ | 30 | 30 |
| ZnCl_2_ | 68 | 68 |
| CoCl_2_ × 6H_2_O | 190 | 190 |
| NiCl2 × 6H_2_O | 24 | 24 |
| Na_2_MoO4 × H_2_O | 36 | 36 |
| FeCl_2_ × 4H_2_O | 14900 | 14900 |
| Na_2_-EDTA | 5200 | 5200 |
| **Trace element solution 2 (TES2)** | **TES2** |  |
| NaSeO_3_ × 5H_2_O | 3 |  |
| Na_2_WO_4_ × 2H_2_O | 8 |  |
| NaOH | 400 |  |

**Table S2. Substrate utilization by the isolated strains**. "Methylacidiphilum infernorum" strain V4 was included for comparison, based on data from reference [1].

| **Substrate** | **Concentration** | **Growth** | | | |
| --- | --- | --- | --- | --- | --- |
|  |  | **IT5** | **IT6** | **B4** | **V4** |
| Methane | 10% (v/v) | + | + | + | + |
| Methanol | 10 mM | + | + | + | + |
| Formate | 20 mM | + | + | − | − |
| Nitromethane | 5 mM | − | − | − | nt |
| Ethane | 10% | − | − | − | nt |
| Ethanol | 5mM | − | −* | − | − |
| Lactaldehyde | 10 mM | − | − | − | nt |
| Acetate | 5 mM | − | − | − | − |
| Propane** | 2.50%, 25 % (v/v) | − | − | − | nt |
| 1-Propanol | 10 mM | − | − | − | − |
| 2-Propanol | 10 mM | + | + | − | nt |
| Acetone | 10 mM | + | + | − | nt |
| Acetol | 10 mM | + | + | − | nt |
| 1,2-Propanediol | 10 mM | − | + | − | nt |
| Methylglyoxal | 1-5 mM | − | − | − | nt |
| Methyl acetoacetate | 5 mM | − | − | − | nt |
| Methyl acetate | 5 mM | − | − | − | nt |
| Glycerol | 5 mM | − | − | − | − |
| 2-Nitropropane | 5 mM | − | − | − | − |
| Butane | 10% | − | − | − | nt |
| 1-butanol | 5 mM | − | − | − | nt |
| 2-butanol | 5 mM | − | − | − | nt |
| Butyraldehyde | 5 mM | − | − | − | − |
| Butanone | 5 mM | − | − | − | nt |

Growth is indicated as: +, growth to OD_600_ > 0.1 after one-week incubation. −, growth to OD_600_ < 0.01 in one month. Concentrations in % are given as volume per volume (v/v). *, weak growth (OD_600_ = ca. 0.05) in one month. nt, not tested. Tested negative: 0.05% (w/v) of Ascorbate, Benzoate, Citrate, Galactose, Gluconate, Glucose, Lactose, Mannose, Pyruvate, Succinate, Sucrose, Xylose, Yeast extract, Methyl methanesulfonate, and 1-Butanesulfonate. The substrates were tested at pH 4.5 and incubated at 50 °C with shaking at 200 rpm. **, propane was tested at oxygen-replete and limited conditions.

**Table S3.** Genomic properties of the isolated strains. Genomic properties of strains V4 and Phi based on data from Hou *et al*. [2] and Erikstad *et al*. [3] were included for comparison.

|  | **"Methylacidiphilum"** | | | |  | **"Methylacidimicrobium"** |
| --- | --- | --- | --- | --- | --- | --- |
| **Features** | **IT5** | **IT6** | **Phi** | **V4** |  | **B4** |
| Size (bp) | 2,185,534 | 2,254,698 | 2,337,855 | 2,287,145 |  | 2,371,269 |
| Contigs | 1 | 1 | 231 | 1 |  | 1 |
| GC % | 44.49 | 40.7 | 41.38 | 45.48 |  | 63.59 |
| Contig N50 | 2,185,606 | 2,254,698 | 64,983 | 2,287,145 |  | 2,371,269 |
| rRNA | 3 | 3 | 3 | 3 |  | 3 |
| tRNA | 46 | 46 | 50 | 46 |  | 46 |
| Other RNA (ncRNAs) | 3 | 3 | 3 | 3 |  | 4 |
| Genes (total) | 2016 | 2079 | 2,259 | 2107 |  | 2221 |
| CDS (total) | 1964 | 2,027 | 2,018 | 2,055 |  | 2168 |
| *pmoCAB* operon | 3 | 3 | 3 | 3 |  | 2 |
| [NiFe] hydrogenase type (1b, 1d, 1h, 3b) | 0, 1, 0, 1 | 0, 1, 0, 1 | 0, 1, 0, 1 | 0, 1, 0, 1 |  | 1, 0, 0, 1 |
| Methanol dehydrogenase (*xoxF, mxaF*) | 1, 0 | 1, 0 | 1, 0 | 1, 0 |  | 2, 0 |
| Nitrogen fixation gene (*nifH*) | 1 | 1 | 1 | 1 |  | 1 |
| Serine cycle key genes (*mclA*, *hpr*) | 0, 0 | 0, 0 | 0, 0 | 0, 0 |  | 0, 0 |
| CO_2_ fixation genes *(cbbL, cbbS*) | 1, 1 | 1, 1 | 1, 1 | 1, 1 |  | 2, 2 |
| RuMP pathway key genes (*hxlA, hslB*) | 0, 0 | 0, 0 | 0, 0 | 0, 0 |  | 0, 0 |
| ANI to strain (V4, Phi) (%) | 96.25, 77.03 | 77.47, 98.36 | 77.72, 100 | 100, 77.72 |  | ND |
| AAI to strain (V4, Phi) (%) | 96.75, 81.39 | 81.67, 98.31 | 98.31, 100 | 100, 98.31 |  | ND |
| *d*DDH to strain (V4, Phi) (%) | 68.4, 19.80 | 20.1, 86.40 | 20.1, 100 | 100, 20.1 |  | ND |
| GenBank Accession | CP065956 | CP065957 | LXQC01 | CP000975 |  | CP066203 |

**Table S4.** Key genes involved in methane oxidation and energy metabolism in strains IT5 and IT6 and their expression. Expression of the genes in cells of strain IT6 grown with various substrates and differential expressions are shown. **Supplied as a separate excel file.**

**Table S5.** Genes common in strains IT5 and IT6 but distinct from strain V4. **Supplied as a separate excel file.**

**Table S6.** Expression level of housekeeping genes of "Methylacidiphilum sp. " IT6 grown under different substrates and used to analyze the robustness of the transcriptome data. **Supplied as a separate excel file.**

**Table S7.** Genes significantly upregulated (Fold change (FC) ≥ 2; False discovery rate (FDR) < 0.05) in 2-propanol and methane-grown cells**. Supplied as a separate excel file.**

**Table S8.** Identification of proteins of the C3 compounds-utilization gene cluster in publicly available verrucomicrobial methanotroph genomes.

**Table S9:** RNA-Seq analysis of "Methylacidiphilum sp." IT6 grown on methane, 2-propanol, acetone, and acetol. **Supplied as a separate excel file.**

**Supplementary Figures**

**
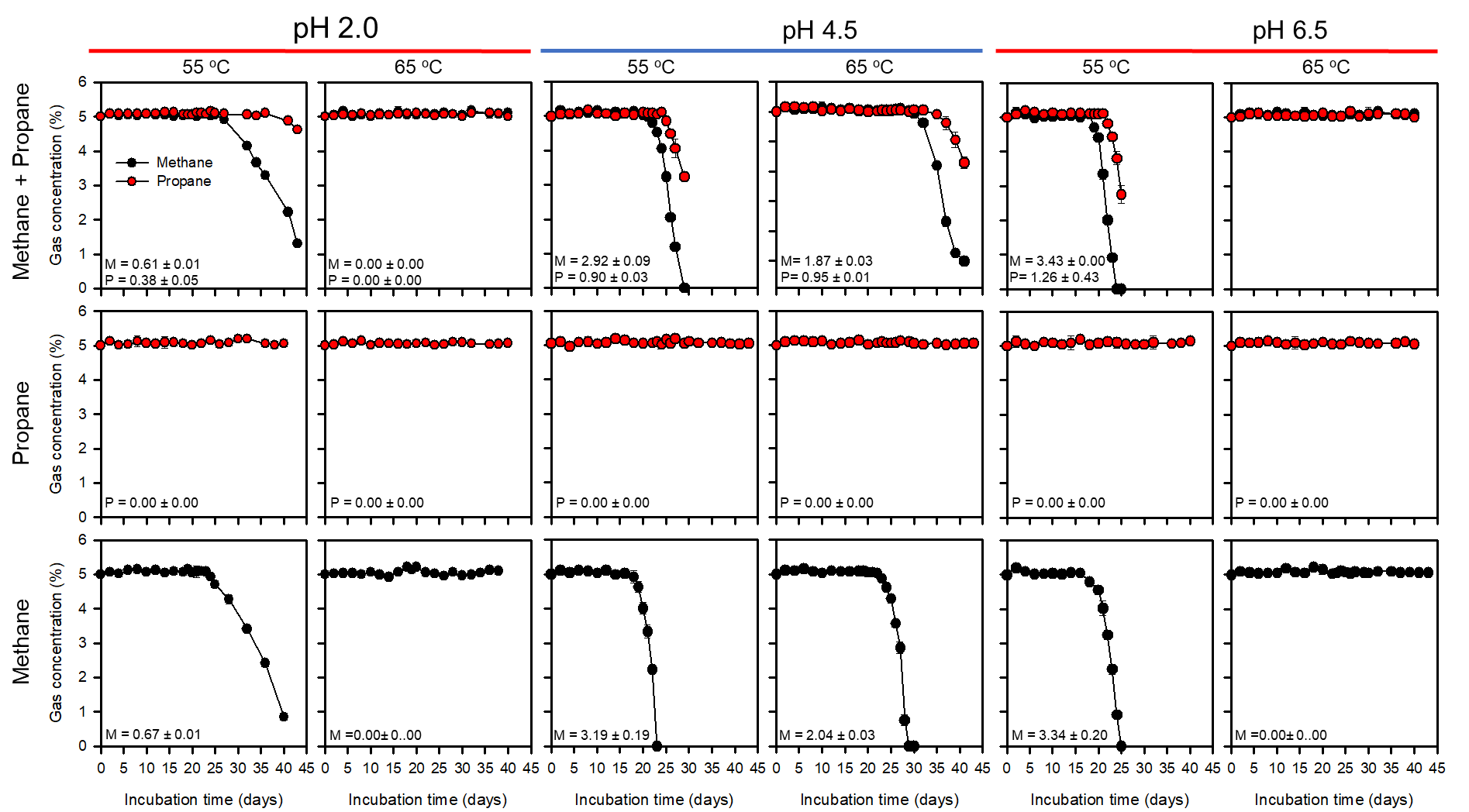
**

**Fig. S1| See next page for the caption.**

**Fig. S1| Consumption of methane and propane over time in enrichment cultures.** The cultivation was started with a mixed slurry of mud pool samples from Pisciarelli hot springs and incubated at 55 and 65 °C, at pH 2.5, 4.5, and 6.5. The upper panel, middle panel, and lower panel indicate cultures with methane+propane, propane, and methane as growth substrates, respectively. Rates of methane (M) and propane (P) oxidation are reported as µmol/ml/day within the figures. The final pH of the sample set up at pH 6.5 dropped to pH 3.0–4.0, probably due to the presence of sulfur particles in the inoculating samples. Before the substrates were completely oxidized, the active cultures were transferred to a fresh medium for enrichment to avoid activity loss.

**Fig. S2| See next page for the caption.**

**Fig. S2| Relative abundance of the major genera in the microbial community of the mud samples and cultures enriched with methane and methane+propane**. The community composition was obtained by 16S rRNA gene amplicon sequencing analysis. 'M' denotes cultures with methane. 'M/P' denotes cultures with methane plus propane. 'Mud' denotes original samples. '55C' and '65C' denote cultures incubated at 55 °C and 65 °C, respectively.

**
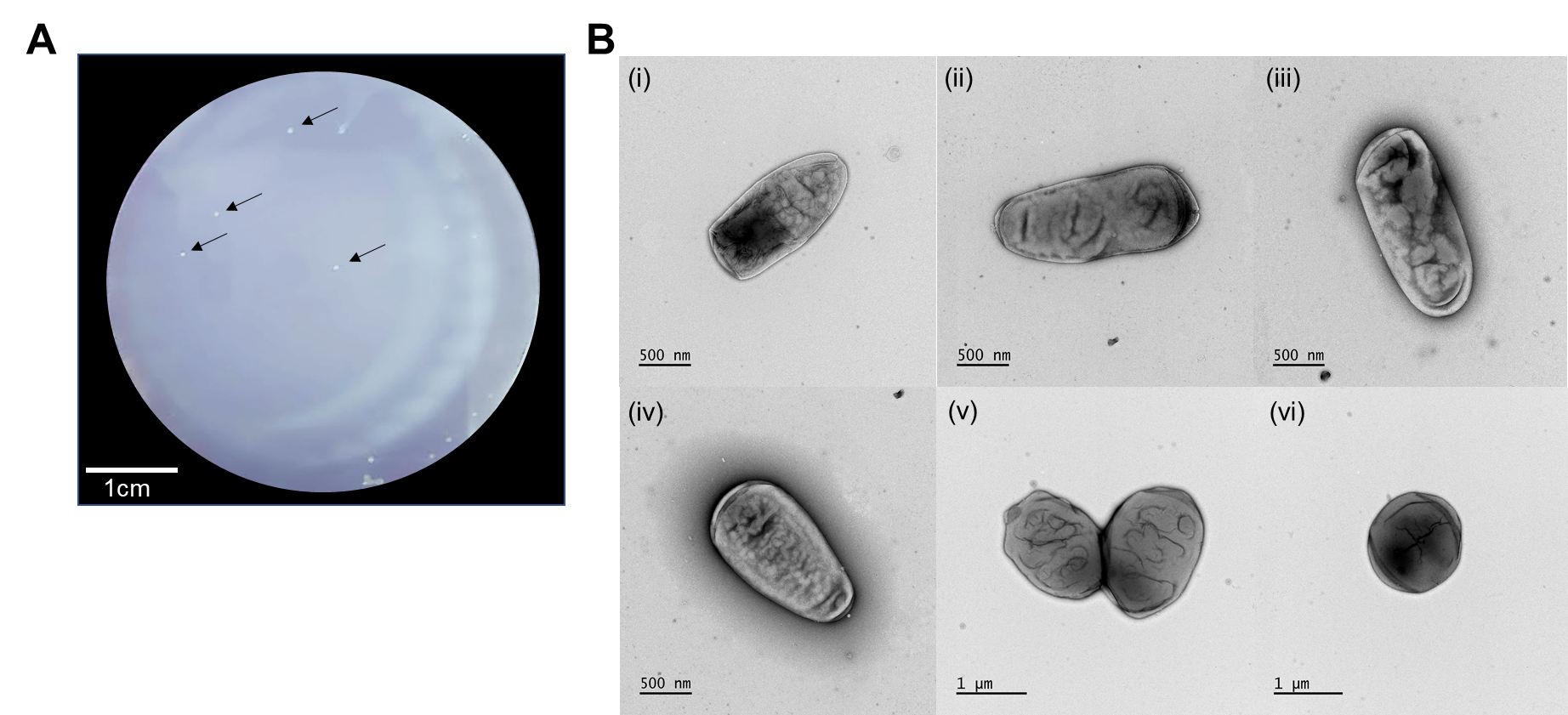
**

**Fig. S3| Morphological properties of isolated verrucomicrobial strains.** (**A**) Small whitish colonies of verrucomicrobial methanotrophs on a polycarbonate membrane filter floated on LSM medium. Black arrows point to some of the colonies. (**B**) Transmission electron microscopy (TEM) images of negatively stained cells of strains, IT6 (i, ii), IT5 (iii, iv), and (v, vi) B4. Bars, 500 nm and 1 µm. The rod-shaped cells observed under the microscope have an average size of 1.2−2.0 µm in length and 0.8−1.3 µm in diameter. Morphological observations were made using cells grown on methane. Cells of exponentially growing cultures of the strains were harvested by centrifugation and applied for TEM analyses at the Korean Basic Science Institute, Ochang Center, Korea, as previously described [4].

**Fig. S4| Effect of CO_2_ (v/v) on the growth of "Methylacidiphilum sp." strain IT6**. (**A**) methanol (30 mM) and (**B**) 2-propanol (10 mM) were supplied as a sole growth substrate. CO_2_ was augmented to the gas phase in the range of 0–10% (v/v). Error bars (invisible if smaller than the symbol size) represent ±1 standard deviation for n ≥ 3 biological replicates.

**
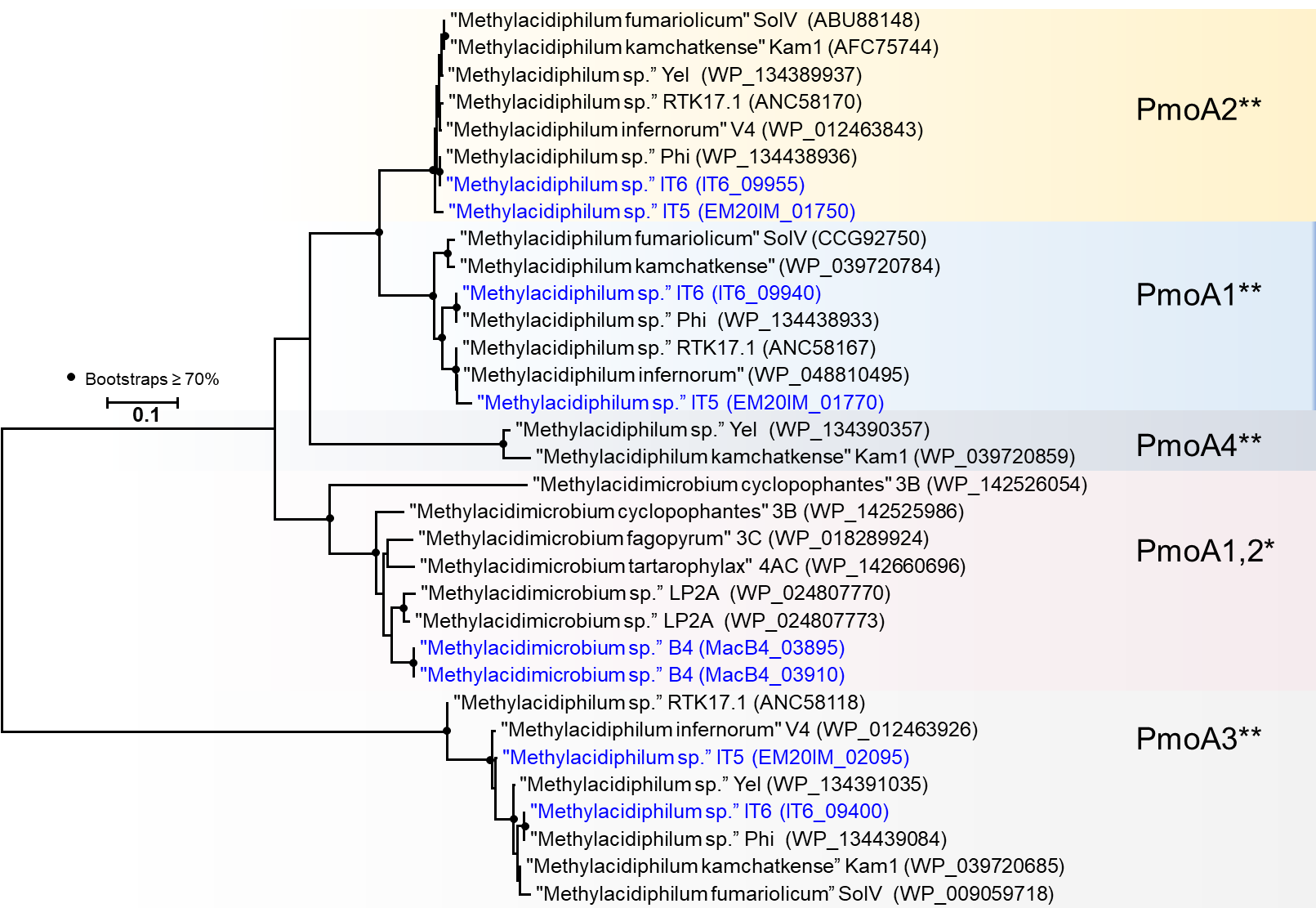
**

**Fig. S5| See next page for the caption.**

**Fig. S5| Phylogenetic tree showing the positions of PmoA1, PmoA2, and PmoA3 of the isolated strains**. The tree was constructed in MEGA7 using the neighbor-joining method, and evolutionary distance was computed using the Dayhoff matrix-based methods with 1000 bootstrap replicates as shown at the branches. Only bootstrap values equal to or greater than 70% are displayed as black circles. The bar represents a 10% estimated-sequence divergence. All ambiguous positions were removed for each sequence pair. There was a total of 252 positions in the final dataset. '**' represents "Methylacidiphilum spp." while '*' represents "Methylacidimicrobium spp.". The names of the isolated strains are in blue, and the numbers in parentheses are the sequence accession number or locus tag in GenBank.


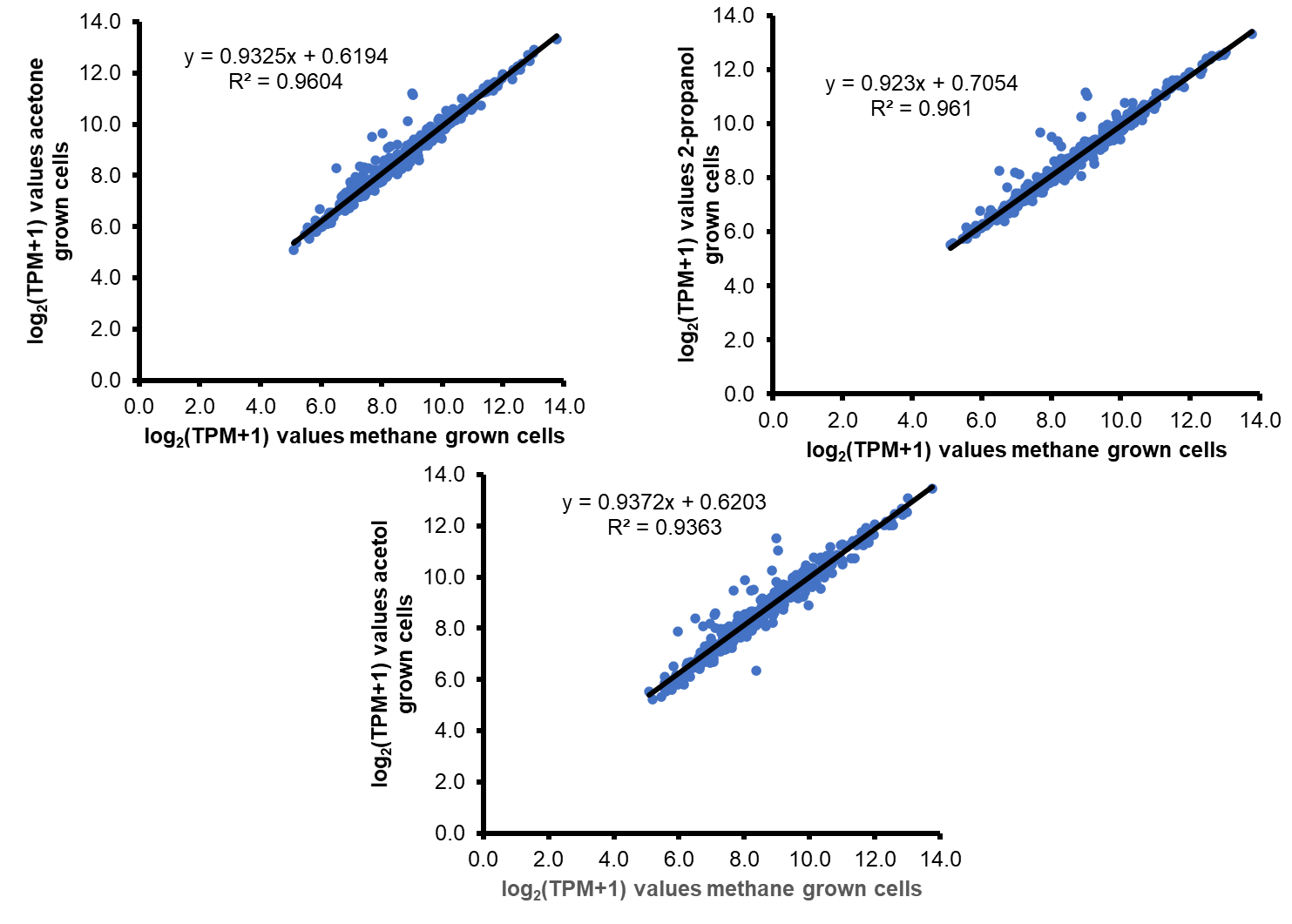


**Fig. S6| See next page for the caption.**

**Fig. S6| Plots of Log_2_(TPM+ 1) values of a group of selected 384 housekeeping genes in strain IT6 with a total size of 428 kbp**. The genes selected are used for energy metabolism with the exception of *pmo*, carbon assimilation (CBB cycle), ribosome assembly, synthesis (amino acid, cell wall, and tRNA), and DNA replication, translation, and transcription. The quality of our transcriptome data was assessed by plotting the logarithmic values of (TPM + 1) of each condition (in triplicates) against each other. From this, we obtained correlation coefficient values of 0.96, 0.96, and 0.93 for methane/2-propanol, methane/acetone, and methane/acetol, respectively.


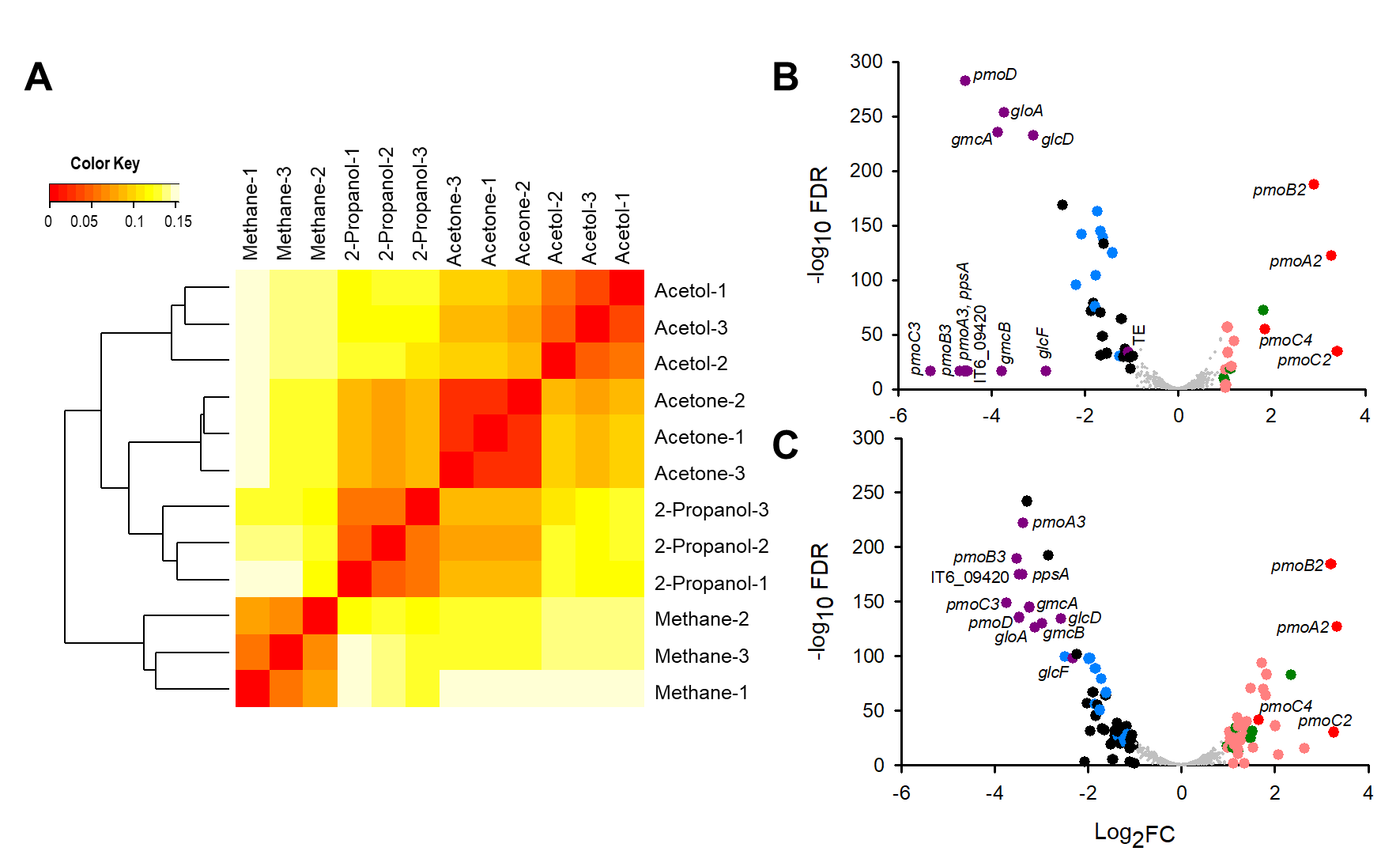


**Fig. S7| Comparative gene expression pattern of cells of strain IT6 grown on C1- and C3-substrates.** (**A**) UPGMA clustering and heatmap of the gene expression profile of cells of strain IT6 grown on methane, 2-propanol, acetone, and acetol. The transcriptome experiments were conducted on cells of strain IT6 grown under an oxygen replete atmosphere for methane and C3-substrates. The clustering was constructed based on Bray-Curtis dissimilarity. The color scale reveals the degree of correlation of gene expression among each substrate. (**B**) A volcano plot showing the differential gene expression between acetone (left) and methane-grown cells (right) under oxygen-replete conditions. (**C**) A volcano plot showing the differential gene expression between acetol (left) and methane-grown cells (right). Small gray dots represent genes with less than two-fold expression difference (FC ≤ 2 and FDR ≥ 0.05). Large colored dots genes of interest with more than two-fold expression difference (FC ≥ 2 and FDR < 0.05) in C3-substrates- and methane-grown cells with the following description: 1) Purple, blue and black are genes in cluster IT6_09370-09425, TCA cycle genes, and other upregulated genes, respectively, in C3-substrates-grown cells, 2) Red, green and pink are genes involved in methane oxidation, formate oxidation and other processes, respectively, in methane grown-cells. Genes colored purple (cluster IT6_09370-09425) and red (Genes for methane oxidation) are labeled with the gene names or Locus tag.


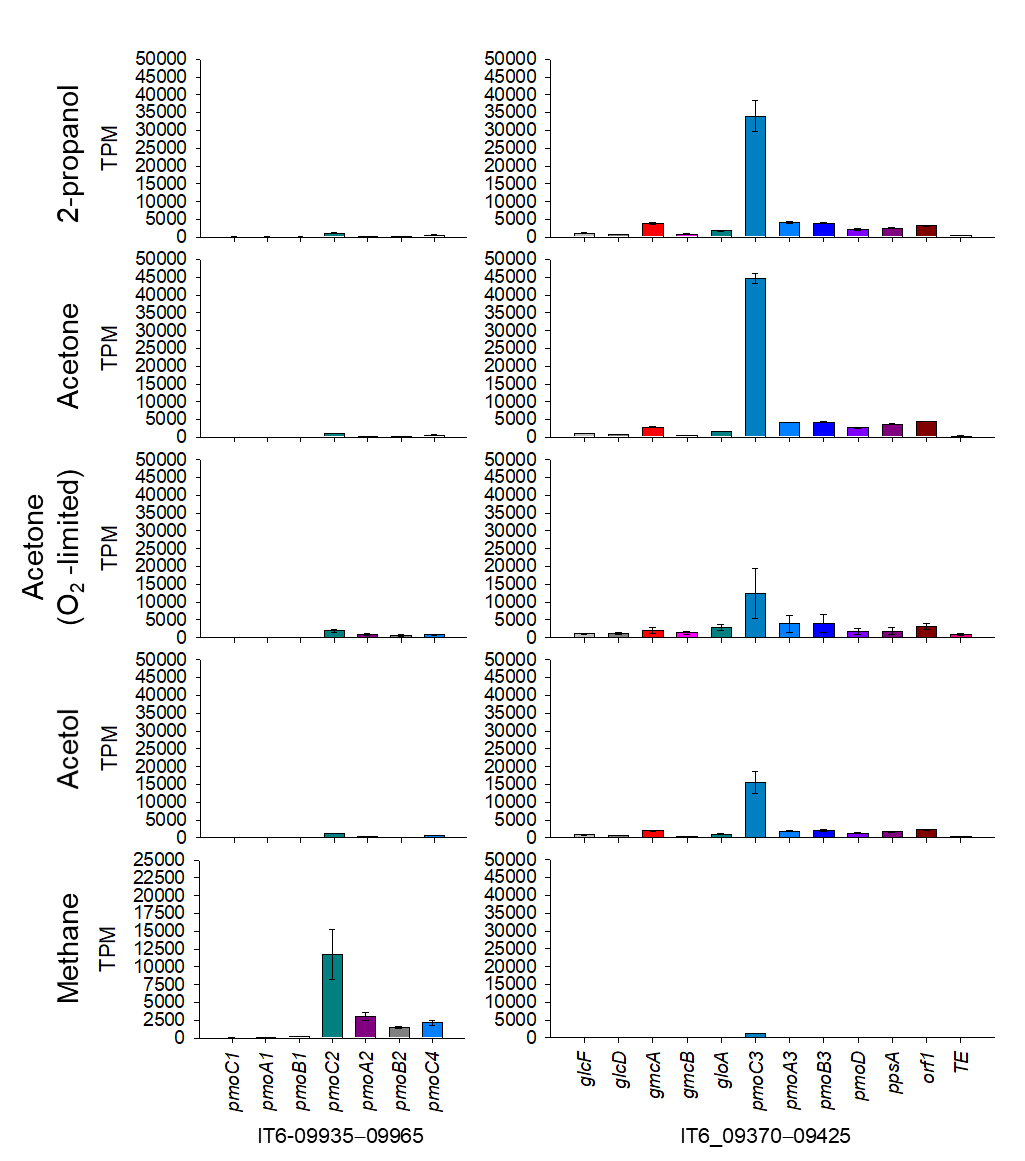


**Fig. S8| Expression (TPM) of the three *pmoCAB* operons encoded in strain IT6 genome in response to growth on methane and C3-substrates.** The expression values of neighboring genes of the *pmoCAB3* operon are also shown. Error bars represent ±1 standard deviation for n ≥ 3 biological replicates


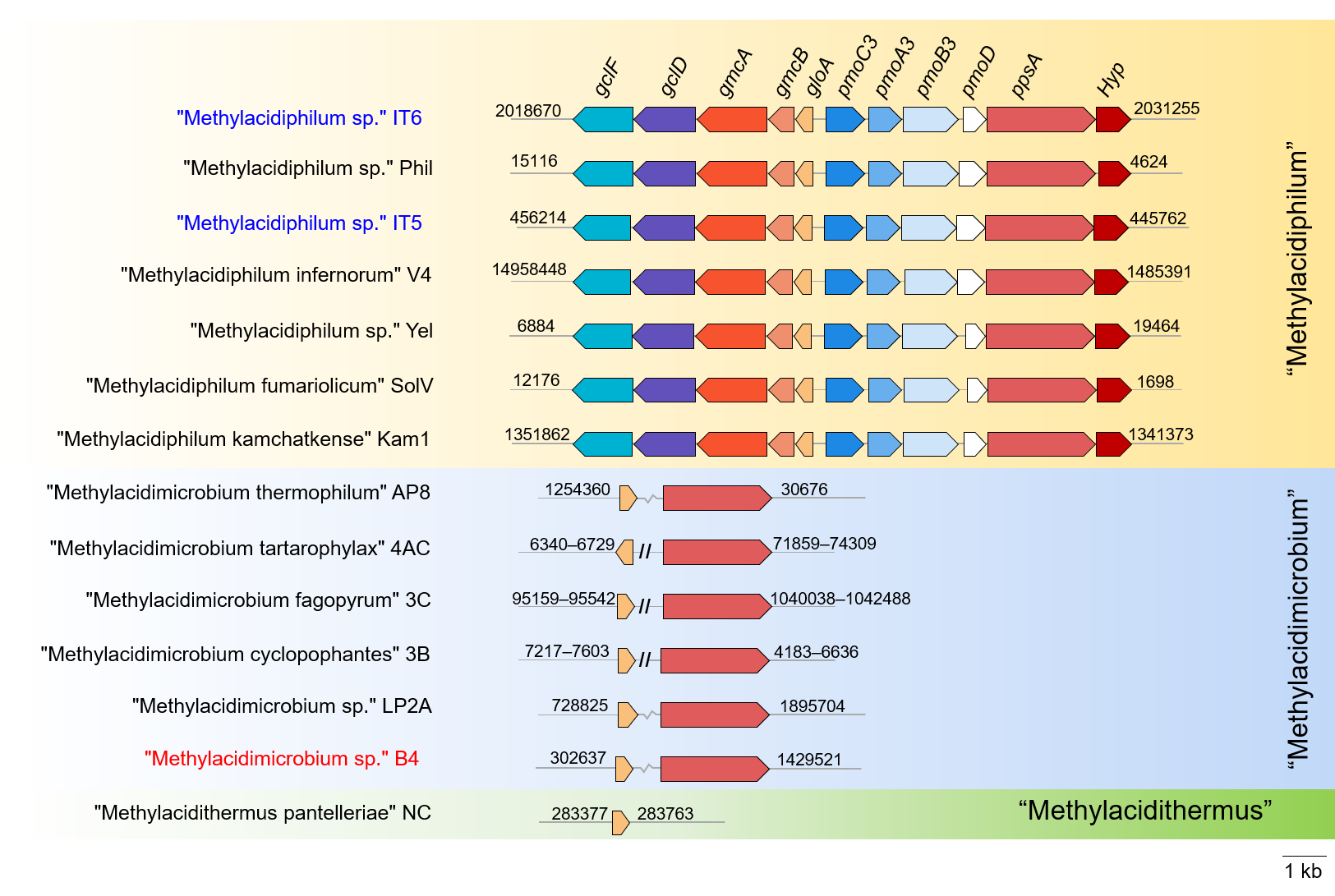


**Fig. S9| Genetic organization of the C3-substrate utilization gene cluster in verrucomicrobial methanotrophs**. Genes are displayed as arrow symbols and are drawn to scale. Genes with similar proteins are shown with the same colors. The gene names are indicated above the genes. Genes with unknown or hypothetical functions are labeled as Hyp. The numbers at the beginning and end of the genomic region indicate the nucleotide positions of the genes in the strain genome. The zigzag line indicates more than 1 kb distance between two genes within the same chromosome. Two slashes separate genes located on different scaffolds. The genome accession numbers/sequence identifiers and detailed information on the genes can be found in **Supplementary Table S8**.

**Fig. S10| See next page for the caption.**

**Fig. S10| Expression of the genes involved in methanol oxidation in strain IT6**. Each bar represents gene expression levels (in transcript per million reads, TPM) for cells grown on methane, 2-propanol, acetone, and acetol, as indicated by the color bar. Expression of the housekeeping genes *gyrA* and *fusA* are shown for comparison. Error bars represent ±1 standard deviation for n ≥ 3 biological replicates.


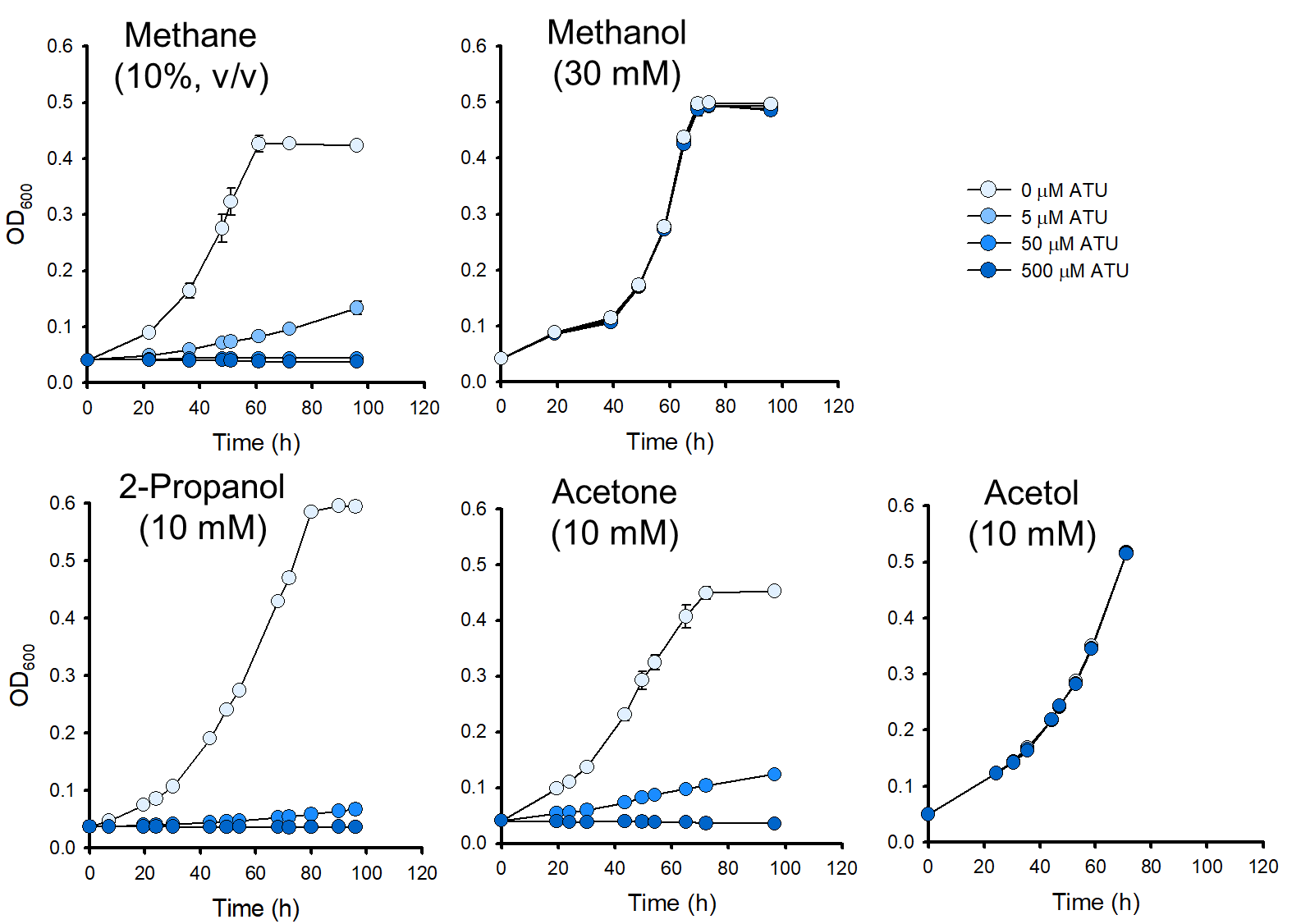


**Fig. S11| See next page for caption.**

**Fig. S11| Inhibitory effect of allylthiourea (ATU), a known CuMMO inhibitor, on the growth of strain IT6**. First, strain IT6 was exposed to varying concentrations of ATU (0, 5, 50, and 500 µM) during growth on methane to determine the threshold level of ATU required for inhibition of growth. Then concentrations of 0, 50, and 500 µM were then applied for growth on C3-substrates. For inoculation, 10% (v/v) of late log phase cells (starting optical density values at 600 nm (OD_600_) < 0.05) were used. The growth of the isolated strains was monitored by measuring changes in the optical density values at 600 nm at intervals. Error bars represent ±1 standard deviation for n ≥ 3 biological replicates.

**
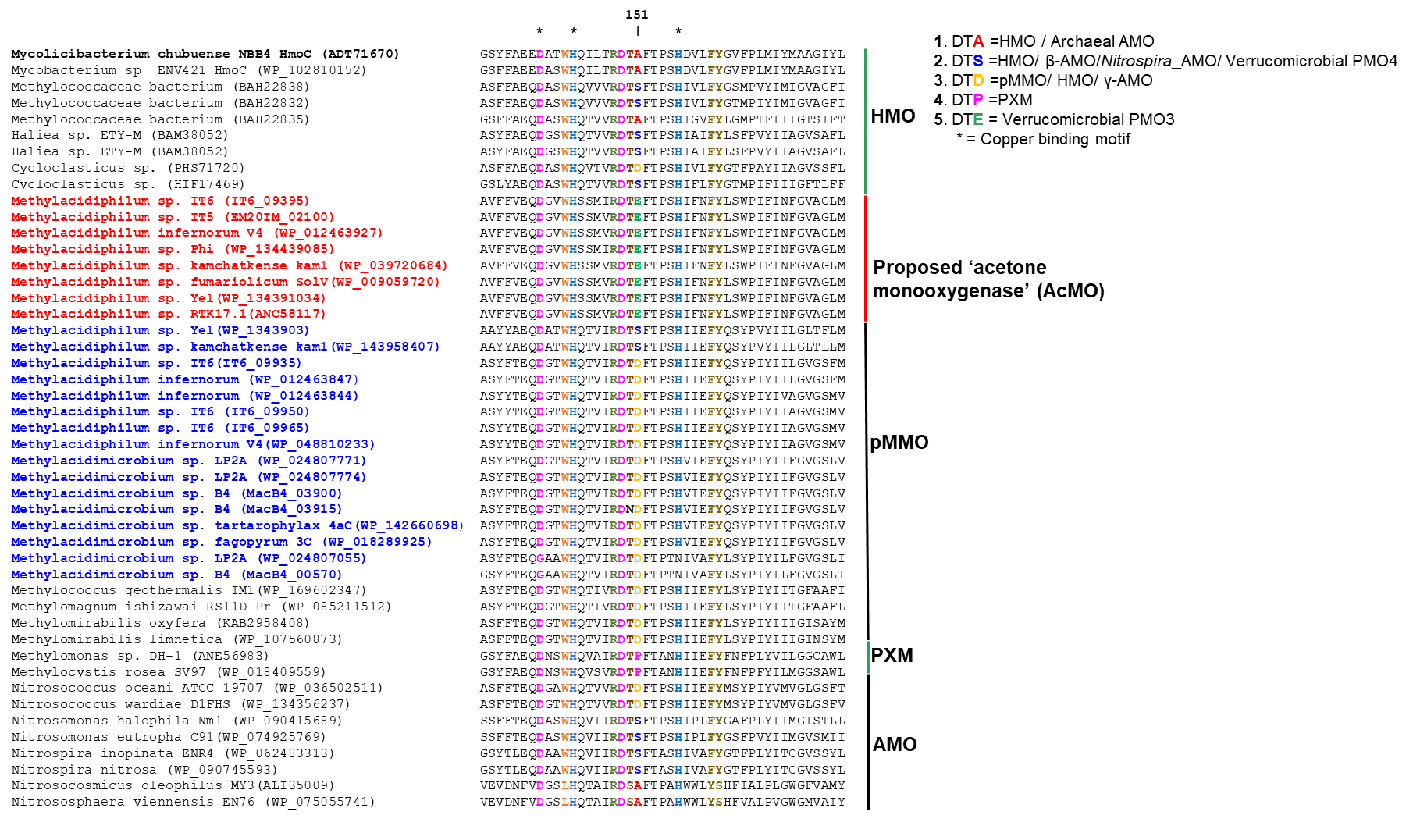
**

**Fig. S12| See next page for the caption.**

**Fig. S12| Potential active site alignments of PmoC with residue 151 highlighted (modified from reference [5]).** The metal-coordinating residues are highlighted by asterisks, and residue 151 is colored according to the amino acid used. The numbers in parentheses are the sequence accession number or locus tag in GenBank.


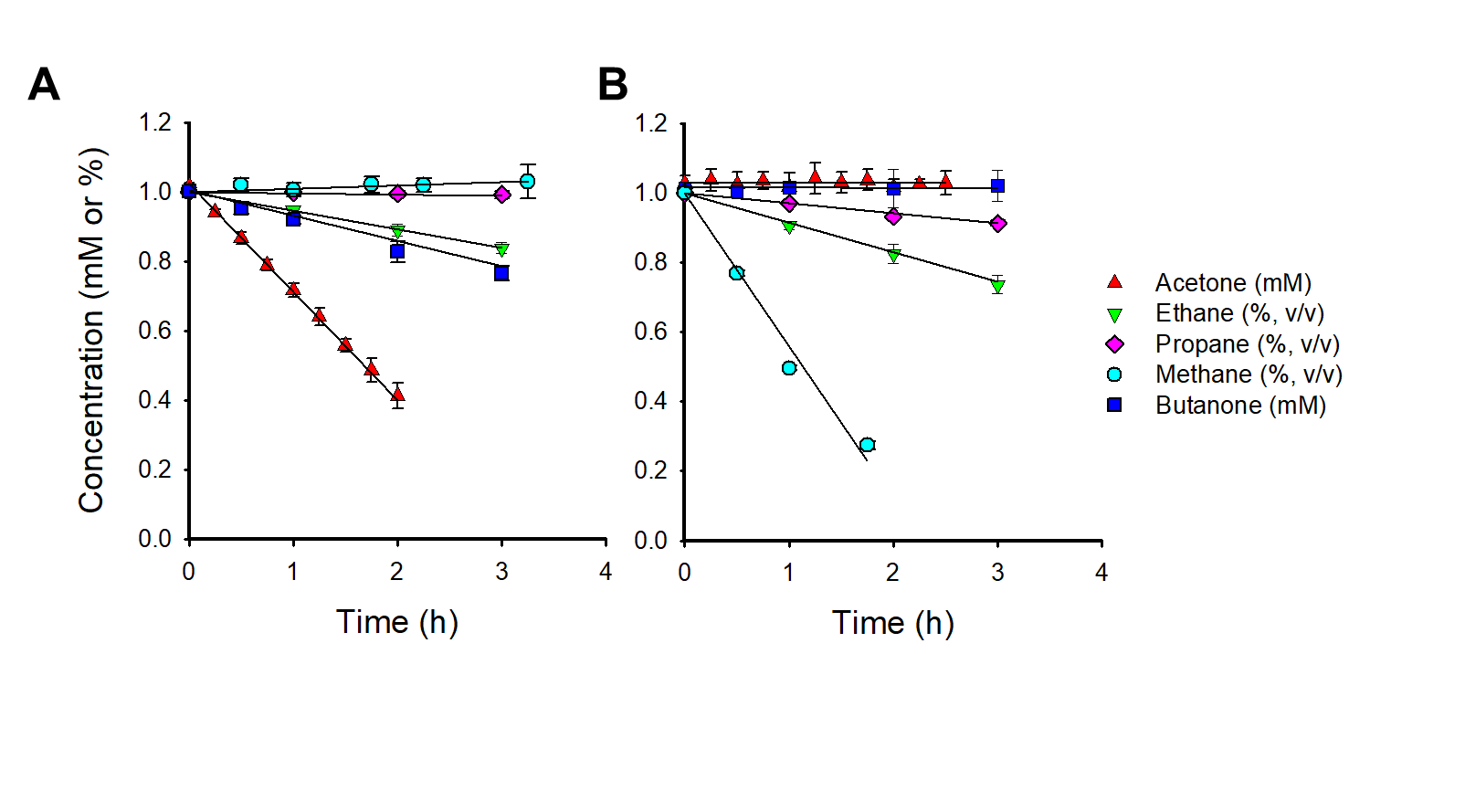


**Fig. S13| Activity of resting cells of strain IT6 towards gaseous hydrocarbons and ketones**. (**A**) Acetone-grown cells and (**B**) methane-grown cells were used. Activity assays with resting cell suspension at OD_600_ =1 were conducted using gaseous and liquid substrates, and the suspensions were incubated at pH 4.5 and 50 °C with shaking at 300 rpm. The data shown are the average of three replicates set up independently, and error bars represent ±1 standard deviation.


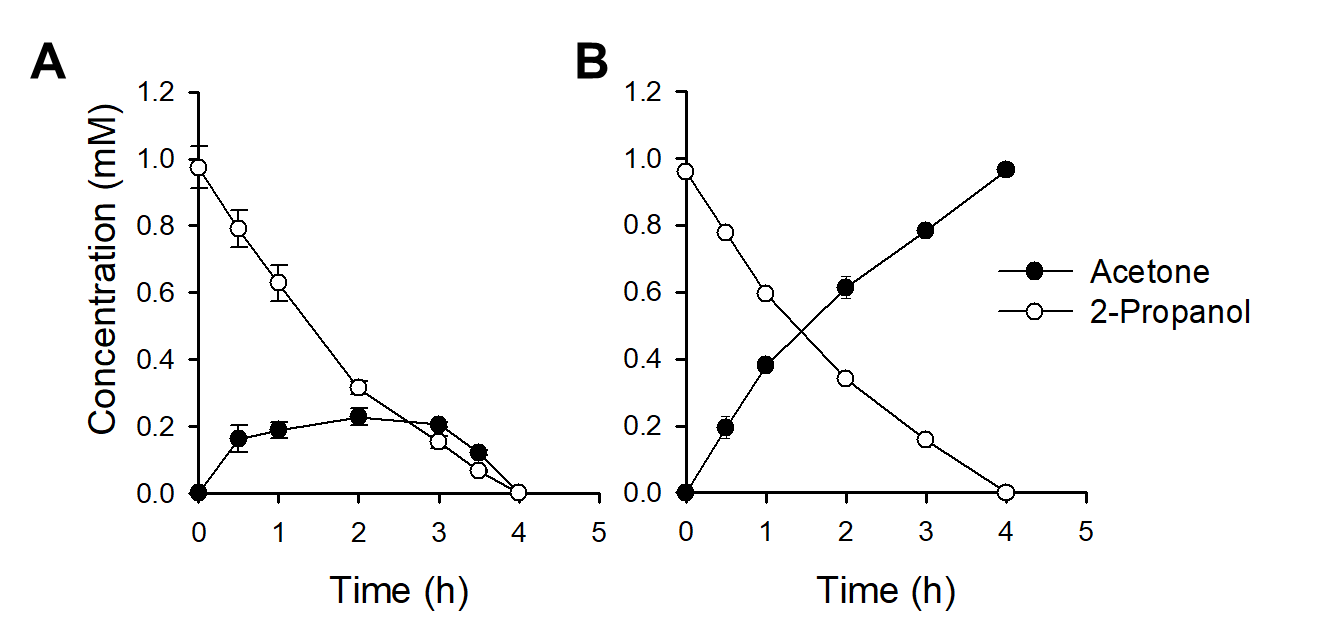


**Fig. S14| Activity of resting cells of strain IT6 grown on 2-propanol towards 2-propanol in (A) the absence of ATU and (B) presence of 500 µM ATU**. The data shown are the average of three replicates set up independently, and error bars represent ±1 standard deviation.


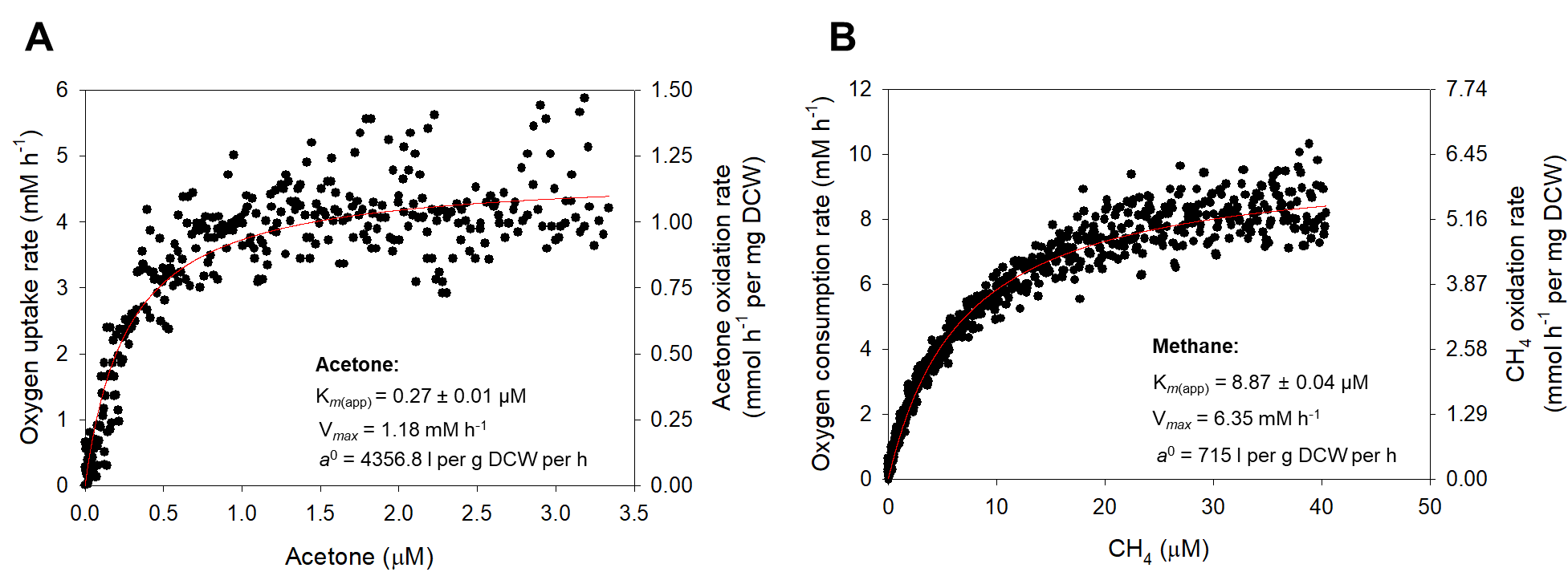


**Fig. S15****| Methane and acetone oxidation kinetics of strain IT6**. (**A**) Acetone-grown cells and (**B**) methane-grown cells were used for the analysis of methane and acetone oxidation kinetics, respectively. Apparent half-saturation constants (K*_m_*_(app)_) and maximum oxidation rates (V*_max_*) for total methane and acetone oxidation were determined by fitting the data to the Michaelis–Menten kinetic equation. The red curve indicates the best fit of the data. Standard errors of the estimates based on nonlinear regression are reported.

**References**

1. Dunfield PF, Yuryev A, Senin P, Smirnova AV, Stott MB, Hou S, et al. Methane oxidation by an extremely acidophilic bacterium of the phylum Verrucomicrobia. Nature. 2007;450:879-82.

2. Hou S, Makarova KS, Saw JHW, Senin P, Ly BV, Zhou Z, et al. Complete genome sequence of the extremely acidophilic methanotroph isolate V4, *Methylacidiphilum infernorum*, a representative of the bacterial phylum Verrucomicrobia. Biol Direct. 2008;3:26.

3. Erikstad HA, Ceballos RM, Smestad NB, Birkeland NK. Global biogeographic distribution patterns of thermoacidophilic Verrucomicrobia methanotrophs suggest allopatric evolution. Front Microbiol. 2019;10:1129.

4. Nguyen N-L, Yu W-J, Yang H-Y, Kim J-G, Jung M-Y, Park S-J, et al. A novel methanotroph in the genus *Methylomonas* that contains a distinct clade of soluble methane monooxygenase. J Microbiol. 2017;55:775-82.

5. Liew EF, Tong D, Coleman NV, Holmes AJ. Mutagenesis of the hydrocarbon monooxygenase indicates a metal centre in subunit-C, and not subunit-B, is essential for copper-containing membrane monooxygenase activity. Microbiology (Reading, England). 2014;160:1267-77.
